# Influence of *Bacillus subtilis* on the corrosion resistance of B30 copper–nickel alloy and the biomass-regulated mineralization mechanism

**DOI:** 10.1128/aem.02286-25

**Published:** 2025-12-10

**Authors:** Meiying Lv, Lixian Chen, Xingyi Tang, Ruoxi Huang, Min Du, Xiyun Zhang, Xingchuan Zhao, Yan Li, Yongxu Du

**Affiliations:** 1 School of Materials Science and Engineering, Liaocheng University 58291, Liaocheng, China; 2 Key Laboratory of Marine Chemistry Theory and Technology, Ministry of Education, College of Chemistry and Chemical Engineering, Ocean University of China 12591, Qingdao, China; Colorado School of Mines, Golden, Colorado, USA

**Keywords:** *Bacillus subtilis*, corrosion inhibition, biomineral layer, biomass, B30 copper–nickel alloy

## Abstract

This study investigates the corrosion inhibition behavior of *Bacillus subtilis* on B30 copper–nickel alloy in seawater, focusing on its biomass components in regulating biomineralization. Results show that *B. subtilis* formed a protective biofilm and induced the precipitation of a uniform biomineral layer, mainly composed of Ca-Mg carbonates. This layer acted as a physical barrier, resulting in a low corrosion current of (5.85 ± 0.08) × 10⁻⁷ A/cm² and reducing the maximum pit depth from 44.74 to 18.54 µm. Furthermore, the roles of different biomass components, such as bacterial cells, extracellular polymeric substances (EPS), and soluble microbial products (SMPs), were also investigated. It was found that all components could initiate mineralization, but with distinct outcomes: bacterial cells primarily served as structural templates; EPS facilitated the formation of highly crystalline and stable Mg-calcite, providing the most durable protection, while SMPs promoted the formation of well-crystallized calcite with comparatively lower protective efficacy.

**IMPORTANCE:**

Corrosion is a critical issue prevalent across various industries, where traditional corrosion control technologies are often limited by high costs, complex implementation, and potential environmental hazards. Biomineralization, as an emerging green anti-corrosion strategy, is not only environmentally friendly but also enables long-term effective protection, reducing reliance on toxic chemical agents and lowering economic costs. However, due to the complexity of microbial systems, the mechanisms underlying biomineralization are not yet fully understood. In this study, different biomass components—including bacterial cells, extracellular polymeric substances, and secreted metabolites—were isolated from *Bacillus subtilis* cultures using a series of separation techniques, and their impacts on the mineralization process were systematically evaluated. This work elucidates the corrosion inhibition mechanism of biomineralization and provides valuable insights into the relationship between specific microbial components and biomineral formation, which holds significant implications for developing eco-friendly corrosion inhibition technologies.

## INTRODUCTION

Corrosion is a major threat to the safety, reliability, and integrity of in-service offshore engineering equipment and infrastructure (1, 2). B30 copper–nickel (Cu–Ni) alloy is widely used in seawater pipelines, heat exchangers, drilling platforms, etc., due to its excellent thermal conductivity, corrosion resistance, machinability, and anti-fouling properties (3). However, it is still difficult to completely avoid corrosion or leakage problems of B30 Cu–Ni alloy in use, which brings huge safety hazards and economic losses (4). It has been reported that some highly corrosive microorganisms colonized on copper alloys and might accelerate the corrosion process by producing corrosive metabolites (sulfides and organic acids) or through extracellular electron transfer with the metal surface (5–7).

Notably, increasing research has revealed that certain microorganisms can also exert beneficial effects during corrosion processes (8, 9). Some microbial species, such as *Bacillus, Pseudomonas*, and *Pseudoalteromonas*, reduced the corrosion rate by several orders of magnitude and served to protect the metals (10–12). The proposed mechanisms by which microbiologically influenced corrosion inhibition include the following: (i) consuming corrosive agents such as dissolved oxygen through aerobic respiration, (ii) secreting antibiotics to kill harmful bacteria, and (iii) forming protective biofilms and mineral precipitates to prevent these agents from diffusing to the metal surface (13). Among these, biofilms (bacterial cells and extracellular secretions) and mineral precipitates form an organic-inorganic hybrid film, exhibiting high and lasting anti-corrosion activity (14). *Bacillus subtilis* (*B. subtilis*) is a facultative aerobic, Gram-positive, and endospore-forming rod bacterium, commonly found in marine environments. Shen et al. (15) investigated the effect of *B. subtilis* on the corrosion behavior of 2A14 aluminum alloy in seawater. The results showed that microbial metabolism produced extracellular polymeric substances (EPS), which contributed to the formation of a biomineralized film composed of calcium and magnesium carbonates (CaMg(CO_3_)_2_), serving as a protective barrier between the alloy matrix and corrosive medium (e.g., Cl⁻ ions). Guo et al. (16) also confirmed the protection of the biomineralized film induced by *B. subtilis*, which inhibited the marine corrosion of steel in a competitive bacterial environment. Biomineralization refers to the process by which ions in solution are converted into biominerals consisting of both organic and inorganic substances under the regulation of living organisms. However, due to the complexity of the microbial system, the corrosion inhibition mechanism is not fully understood, and the specific bacterial components that regulate the morphology, structure, and composition of biominerals have not been clearly defined. In this work, different biomass components of *B. subtilis*, such as bacterial cells, EPS, and secreted metabolites, were isolated from bacterial cultures through a series of separation techniques and utilized to influence the mineralization process. The effects of *B. subtilis* and its biomass on the corrosion of B30 Cu–Ni alloy were investigated through surface analysis and electrochemical measurement techniques. This study aimed to reveal the mechanism of *B. subtilis*-induced corrosion inhibition on B30 Cu–Ni alloy, focusing on the role of biomass in regulating biomineralization.

## MATERIALS AND METHODS

### Coupon preparation and bacterial culture

The B30 Cu–Ni alloy (Ni 29.72, Fe 0.64, Mn 0.75, C 0.02, Si 0.11, S 0.01, remaining Cu, wt.%) used in tests was cut to 10 × 10 × 5 mm, and then sequentially abraded with 400, 600, 800, 1,000, and 2,000 grit silicon carbide sandpaper. All coupons were ultrasonically cleaned with deionized water and anhydrous ethanol, dried with a stream of nitrogen (N_2_), and then sterilized by ultraviolet lamp for 20 min before experiments were carried out.

*B. subtilis* (ATCC 23270) was cultured in 2216E medium with the composition of 5.0 g peptone, 1.0 g yeast extract, 0.1 g ferric citrate, 19.45 g NaCl, 5.98 g MgCl_2_, 3.24 g Na_2_SO4, 1.80 g CaCl_2_, 0.55 g KCl, 0.16 g Na_2_CO_3_, 0.08 g KBr, 0.034 g SrCl_2_, 0.022 g H_3_BO_3_, 0.004 g NaSiO_3_, 0.0024 g NaF, 0.0016 g NH_4_NO_3_, and 0.008 g Na_2_HPO_4_ per liter of natural seawater. The pH of the solution was adjusted to 7.2 ± 0.1 and then autoclaved at 121°C for 20 min. The seed cultures were maintained in a constant‐temperature incubator at 37°C for 72 h under aerobic conditions. The bacterial strain *B. subtilis* was added to experimental systems according to a 5% vol ratio. The growth curve of *B. subtilis* was determined in the test solution using ultraviolet spectrophotometry (UV5100, METASH, Shanghai, China) at an optical density value of 600 nm (OD_600_) in the presence of B30 Cu–Ni alloy. Bacterial adhesion on the metal surface was performed using the plate counting method under a fluorescence microscope (FM, DM2500, Leica, Germany). Before observations, coupons were stained using the 4′,6-diamidino-2-phenylindole (DAPI, 1 µg/mL) in darkness for 15 min.

### Separation and extraction of biomass

After incubation for 72 h, the bacterial inoculum was cryo-centrifuged at 3,000 r/min, the supernatant was collected, and the residual cell debris was removed by a 0.22 µm pore size nitrate cellulose filter to obtain the soluble microbial products (SMPs). Then, the collected bacterial solution was washed twice in 0.9% sterile saline, extracted with 2% EDTA at 4°C for 3 h, centrifuged at 10,000 r/min for 10 min, and the residue was obtained as bacterial cells. The supernatant was filtered by a 0.22 µm filter and placed in a dialysis bag (molecular weight cut-off 3,500 Da) for 24 h to remove extractant residues, inorganic ions, and small molecule interfering components in the solution to obtain EPS. The obtained biomass was refrigerated at 4°C. The crystallization experiment was performed by mixing various biomass with concentrated seawater at room temperature (25°C ± 1°C). A glass substrate was placed at the bottom of a wide-mouth flask to collect the mineral crystals formed from the solution. It was taken out after 72 h, and the crystal products were alternately washed three times with distilled water and ethanol to remove the biological residues that may be adsorbed on the mineral surface and finally dried for subsequent experiments.

### Morphological and compositional characterization

For corrosion evaluation, the B30 Cu–Ni alloy was placed in fresh seawater without bacterial cells and inoculated with *B. subtilis*, respectively. After 3 d and 14 d, coupons were taken out from a set of vials, washed gently with a phosphate-buffered solution (PBS, pH = 7.4), and immobilized in the glutaraldehyde solution (5%) for 2 h. Subsequently, the coupons with biofilm were dehydrated in the 50%, 70%, 90%, and 100% ethanol solutions for 15 min, except for the last step, for 30 min, and then dried with high-purity N_2_. The morphology of the covered surface was characterized by scanning electron microscopy (SEM, Zeiss Ultra 55, Germany) combined with energy-dispersive X-ray spectroscopy (EDS, X-Max, Oxford, Britain) after gold sputter-coating. Furthermore, the coupons covered with corrosion products and biomineralized products were analyzed using X-ray photoelectron spectroscopy (XPS, Thermo Fisher Scientific, USA). The wide range XPS (emission angle 45°, 0–1,200 eV) for binding energy spectra and XPS high-resolution spectra of C1s, Cu 2p, Ni 2p, Mg 2s, and Ca 2p scans were analyzed using monochromatic Al Kα radiation. After that, the coupons were brushed using a soft brush and immersed in a pickling solution containing hexamethylenetetramine for 2 min to remove the biofilm and corrosion products. The pit morphology on the alloy surface, after the removal of corrosion products, was characterized using a 3D digital video microscopy (HiRox RH-8800, Japan). For each coupon, four different areas were measured along a straight line to obtain both the maximum and the average pit depth. Furthermore, the precipitated products in the biomass medium were collected for morphology and crystal structure analysis with SEM (JSM–6700F, Japan), X-ray powder diffraction (XRD, Tongda TD-3700, China) using a Cu Kα target with a scanning rate of 5°/min, and high-resolution transmission electron microscopy (HRTEM, FEI Tecnai G2, USA) combined with selected area electron diffraction (SAED). The molecular structures of biomass and crystal products were analyzed using Fourier transform infrared (FT-IR, Thermo Fisher Nicolet 6700, USA) spectroscopy. The weight percentage of mineralized precipitation of each biomass was determined through thermogravimetric analysis (TGA, Netzsch STA-449 F5, Germany). The test was carried out under a N_2_ atmosphere with a heating rate of 10°C/min in the 30°C–800°C temperature range.

### Corrosion measurements

The electrochemical characteristics of B30 Cu–Ni alloy were studied using an electrochemical workstation (VSP-300, Bio-Logic SAS, France). B30 Cu–Ni alloy with an exposed area of 1 cm^2^ was employed as the working electrode (WE). Platinum sheet and saturated calomel electrode were used as the counter electrode (CE) and reference electrode (RE), respectively. After the open circuit potential (OCP) reached a steady-state value, electrochemical impedance spectroscopy (EIS) was measured by scanning the frequency range between 10^–2^ Hz and 10^5^ Hz, and the amplitude of a sinusoidal voltage signal was 10 mV. Potentiodynamic polarization curves were monitored at the sweep rate of 0.5 mV/s with the potential range from −500 to +500 mV vs. OCP. The impedance data and polarization curves were analyzed using the Zview and Cview software (Scribner Inc.). All experiments were performed at 25°C and repeated at least three times.

## RESULTS

### Bacterial growth

**Fig 1:**
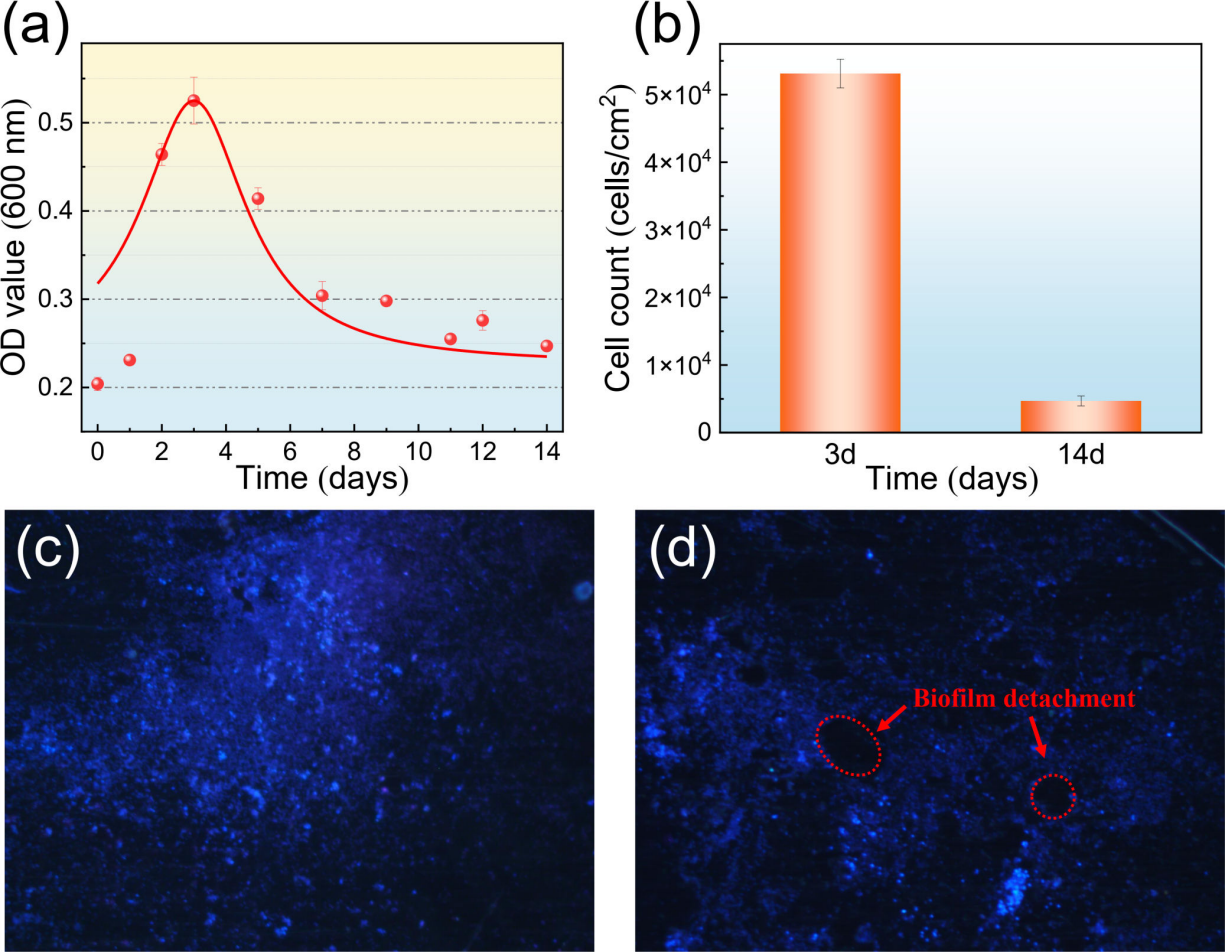
The growth curve of planktonic *B. subtilis* at OD_600_ (**a**), the sessile cell counts of *B. subtilis* on the surface of B30 Cu–Ni alloy (**b**), and the fluorescence images of *B. subtilis* biofilm after immersion for 3 d (**c**) and 14 d (**d**).

Figure 1 shows the growth curve of planktonic *B. subtilis*, as well as the sessile cell counts on the surface of B30 Cu–Ni alloy, and the fluorescence images of *B. subtilis* biofilm. Figure 1a illustrates the bacterial growth, with cell numbers peaking on the 3 d and then decreasing due to nutrient depletion. *B. subtilis* mainly used organic substrates as its energy source, and the limited nutrient resources in the test solution could not continue to support more bacterial cells. This led to *B. subtilis* entering the decline phase, with live cells rapidly decreasing. As indicated in Fig. 1b, the sessile cell counts on B30 Cu–Ni alloy on the 3 d were also significantly higher than that on the 14 d, consistent with the trend shown in Fig. 1a. The corresponding fluorescence images confirmed the attachment of the bacteria to the surface of B30 Cu–Ni alloy (indicated by blue dots in Fig. 1c), and the formation of a dense biofilm on the 3 d. After 14 d, nutrient depletion led to bacterial activity reduction and partial biofilm detachment (Fig. 1d).

### Surface morphology and composition analysis

**Fig 2:**
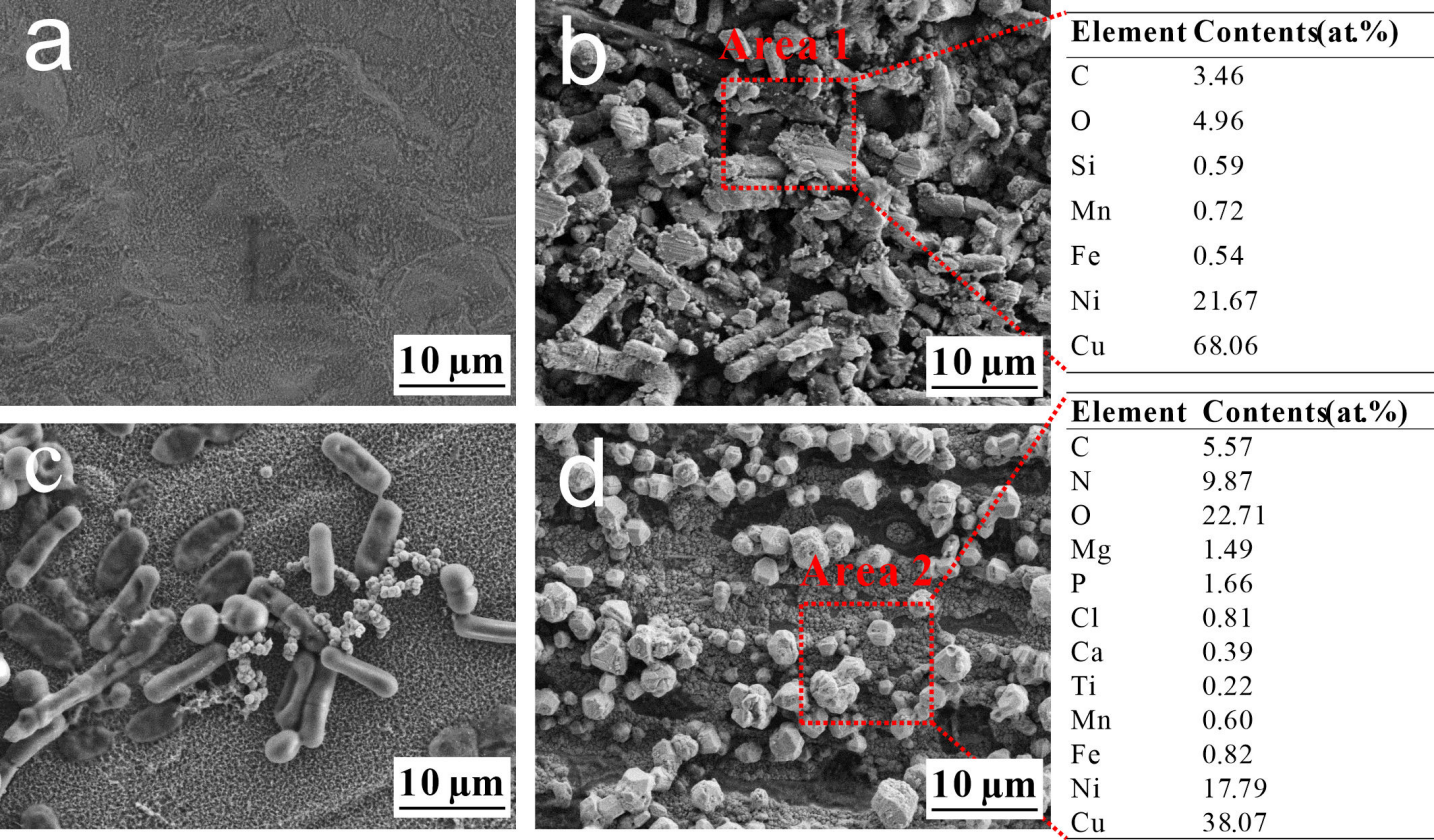
SEM images of the B30 Cu–Ni alloy after immersion in different media: sterile for 3 d (**a**), sterile for 14 d (**b**), *B. subtilis* for 3 d (**c**), *B. subtilis* for 14 d (**d**), and the corresponding EDS data derived from Area 1 and Area 2.

Figure 2 shows the biofilm and corrosion products morphology on B30 Cu–Ni alloy after immersion in sterile and *B. subtilis* media for 3 and 14 d, as well as the corresponding EDS elemental composition. In the sterile system for 3 d, the coupon formed a thin and dense corrosion product film (Fig. 2a). With the extension of the immersion time, the film layer appeared to be uneven and porous (Fig. 2b), and EDS analysis exhibited the predominance of Cu, Ni, and O elements, suggesting the formation of representative corrosion products such as Cu_2_O, CuO, and NiO (7). In the biological system, there were numerous cells attached to the surface, indicating the good growth of *B. subtilis* on the B30 Cu–Ni alloy (Fig. 2c). After 14 d, the alloy surface was uniformly covered with a layer composed of irregularly rhombohedral particles (Fig. 2d), a crystal habit of carbonate minerals such as calcite (17). No sessile cells were observed, which might be attributed to their covering and embedding within the mineral deposits and corrosion products (18). The consistent observations from multiple random regions confirmed the formation of these mineral particles and their characteristic morphology (Fig. S1). Furthermore, EDS analysis results reflected the distribution of C (5.57%), N (9.87%), and P (1.66%) elements on the surface, serving as the indicator of bacterial biomass from cells and the biofilm (19). The elements of Ca and Mg were only present in the biological system, suggesting that plenty of mineral particles were closely associated with the *B. subtilis*, inducing the formation of Ca-Mg carbonates after incubation for 14 d (20).

**Fig 3:**
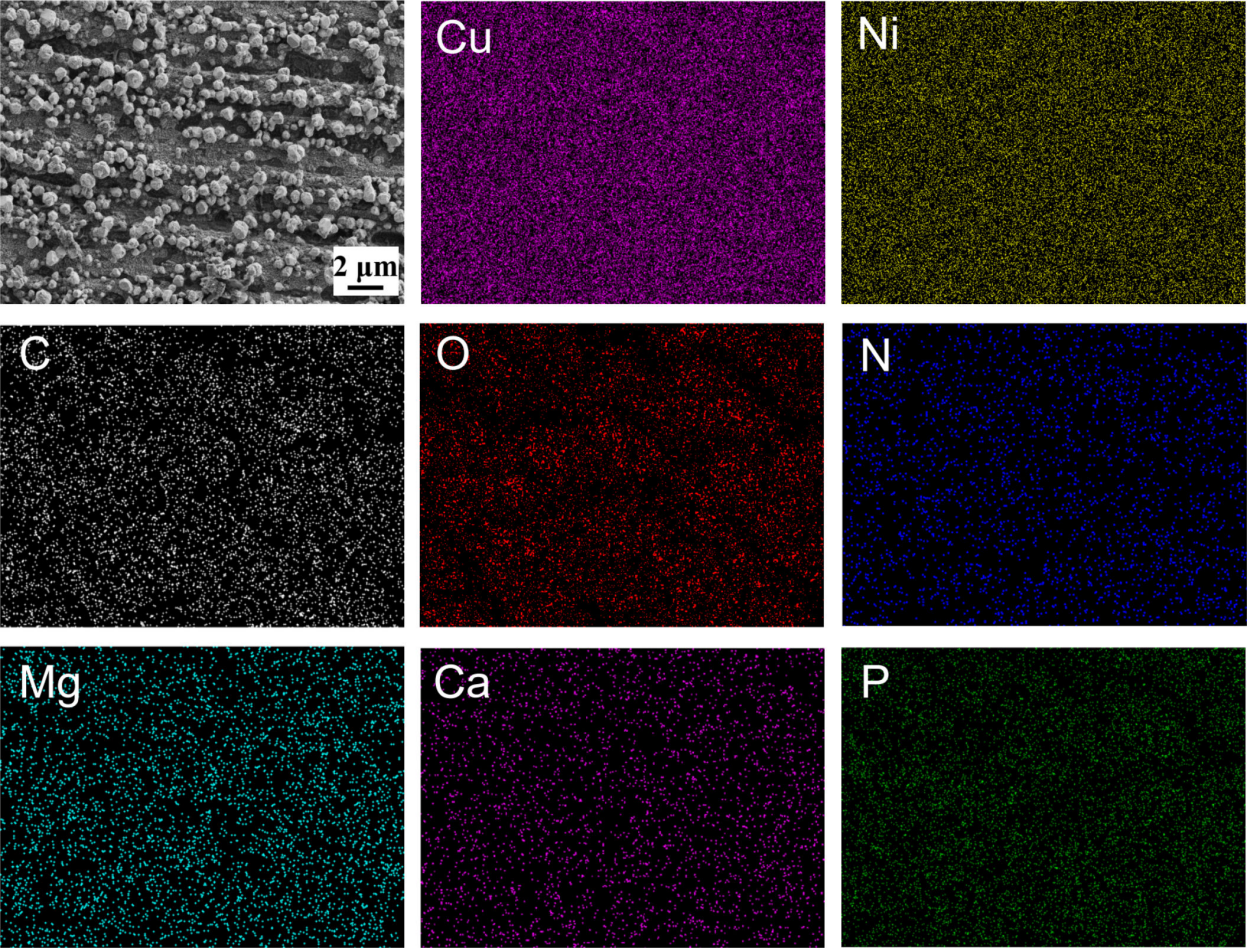
Elemental mapping images of Cu, Ni, C, O, N, Mg, Ca, and P elements for B30 Cu–Ni alloy after immersion for 14 d in the *B. subtilis* medium.

To probe the elemental composition and spatial distribution of the surface layer, elemental mapping was performed on the B30 Cu–Ni alloy after 14 d of immersion in both the *B. subtilis* and the sterile control medium. As shown in Fig. 3, in the *B. subtilis* system, the alloy surface was uniformly covered with a layer composed of C, O, N, Mg, Ca, and P, along with underlying Cu and Ni signals. In particular, Ca and Mg were homogeneously distributed across the entire surface, confirming the formation of a continuous mineral layer. In contrast, EDS mapping of the coupon from the sterile system (Fig. S2) revealed that the surface was mainly composed of Cu, Ni, O, and a small amount of C elements, and no Ca and Mg signals were detected. It demonstrated that the significant accumulation of Ca and Mg minerals was induced by bacterial activity, rather than spontaneous precipitation from the seawater medium. Furthermore, the presence of C, N, and P with the mineral layer confirmed the crucial role of bacterial biomass (e.g., cells and EPS) in providing nucleation sites and structural templates for the formation of Ca-Mg biominerals (21).

**Fig 4:**
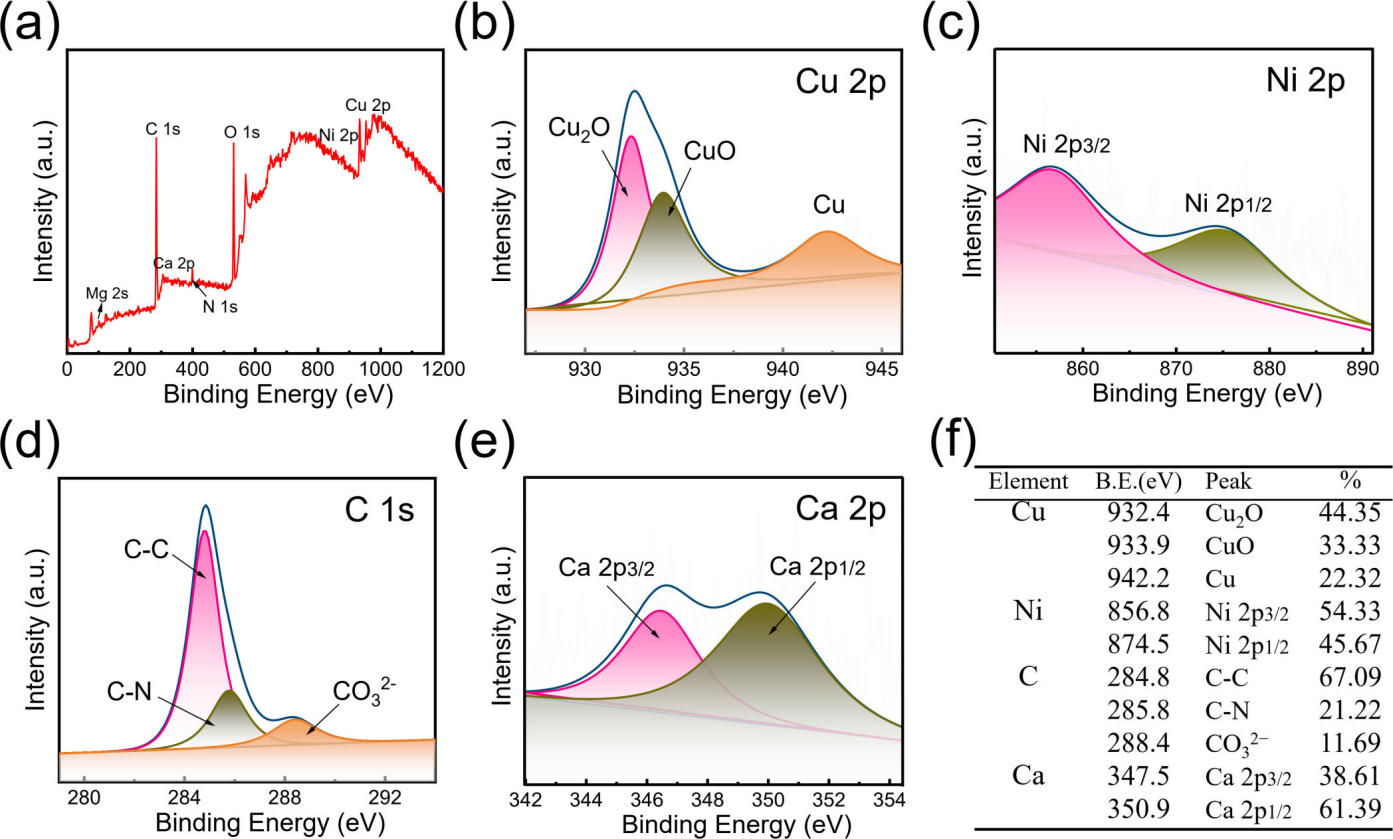
XPS survey spectra (**a**), high-resolution spectra of Cu 2p (**b**), Ni 2p (**c**), C 1s (**d**), and Ca 2p (**e**), and the corresponding surface element peaks attribution (**f**) of the B30 Cu–Ni alloy after 14 d of immersion in the *B. subtilis* system.

Figure 4a shows the XPS survey spectrum of B30 Cu–Ni alloy after 14 d immersion in the *B. subtilis* medium, confirming the existence of Cu, Ni, C, O, N, Ca, and Mg compounds in the surface film. The Ni 2p signal was weak, probably due to its enrichment in the inner layer of the corrosion products. To better understand the elemental valence states within the film layer, the high-resolution XPS spectra of Cu 2p, Ni 2p, C 1s, and Ca 2p were fitted by peaks and the corresponding binding energies of each compound are listed in Fig. 4f. The Cu 2p spectrum of the corrosion products on the surface of B30 Cu–Ni alloy can be fitted with three peaks at 932.4 eV, 933.9 eV, and 942.2 eV (Fig. 4b), which were assigned to Cu_2_O, CuO, and metallic Cu, respectively. Compared with the peak intensity of Cu 2p, the Ni 2p signal in the spectrum was significantly weaker, and the peaks were mainly attributed to oxides and hydroxides of divalent nickel at 856.8 eV and 874.5 eV (Fig. 4c). In the C 1s spectra, the peaks at 284.8 eV, 285.8 eV, and 288.4 eV were attributed to the binding energies of C–C, C–N, and CO_3_^2−^ bonds, respectively (Fig. 4d). Combined with the EDS analysis, the C–C and C–N signals were the result of biofilm formation on the coupon surface, while the CO_3_^2−^ could be attributed to the mineral precipitates of carbonates. Figure 4e presents the high-resolution Ca 2p spectrum, and the peaks at 347.5 eV and 350.9 eV were assigned to calcium carbonate (CaCO_3_) from microbial bio-mineralization, indicating the presence of a biomineral layer on the alloy surface.

**Fig 5:**
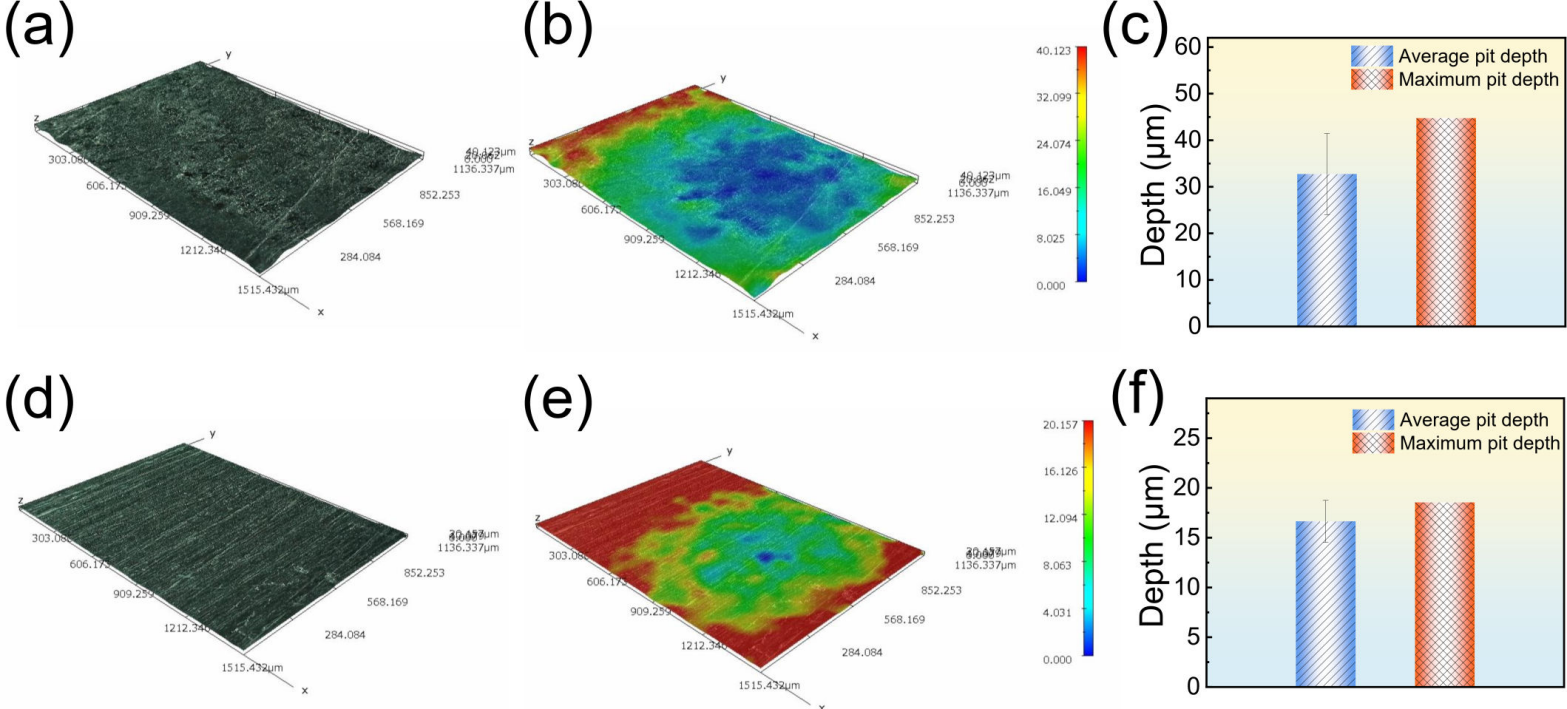
Corrosion morphology (**a and d**), 3D profile (**b and e**), and corrosion pit parameters (**c and f**) of B30 Cu–Ni alloy after 14 d of immersion in sterile (**a–c**) and *B. subtilis* (**d–f**) systems.

The surface morphology of the B30 Cu–Ni alloy after removing corrosion products is shown in Fig. 5. To obtain the pitting data, each coupon was systematically profiled by measuring the pit depth at four areas along a straight line (Fig. S3 and S4). After 14 d of immersion in the sterile system, the surface exhibited severe pitting corrosion with an average pit depth of 32.73 ± 8.72 µm and a maximum pit depth of 44.74 µm. In contrast, the coupon exposed to the *B. subtilis* system showed a markedly improved surface state, with the original machining scratches still partially visible. The average pit depth was significantly reduced to 16.64 ± 2.11 µm, and the maximum pit depth was limited to 18.54 µm, indicating the effective inhibition of localized corrosion by *B. subtilis*.

### Electrochemical analysis

**Fig 6:**
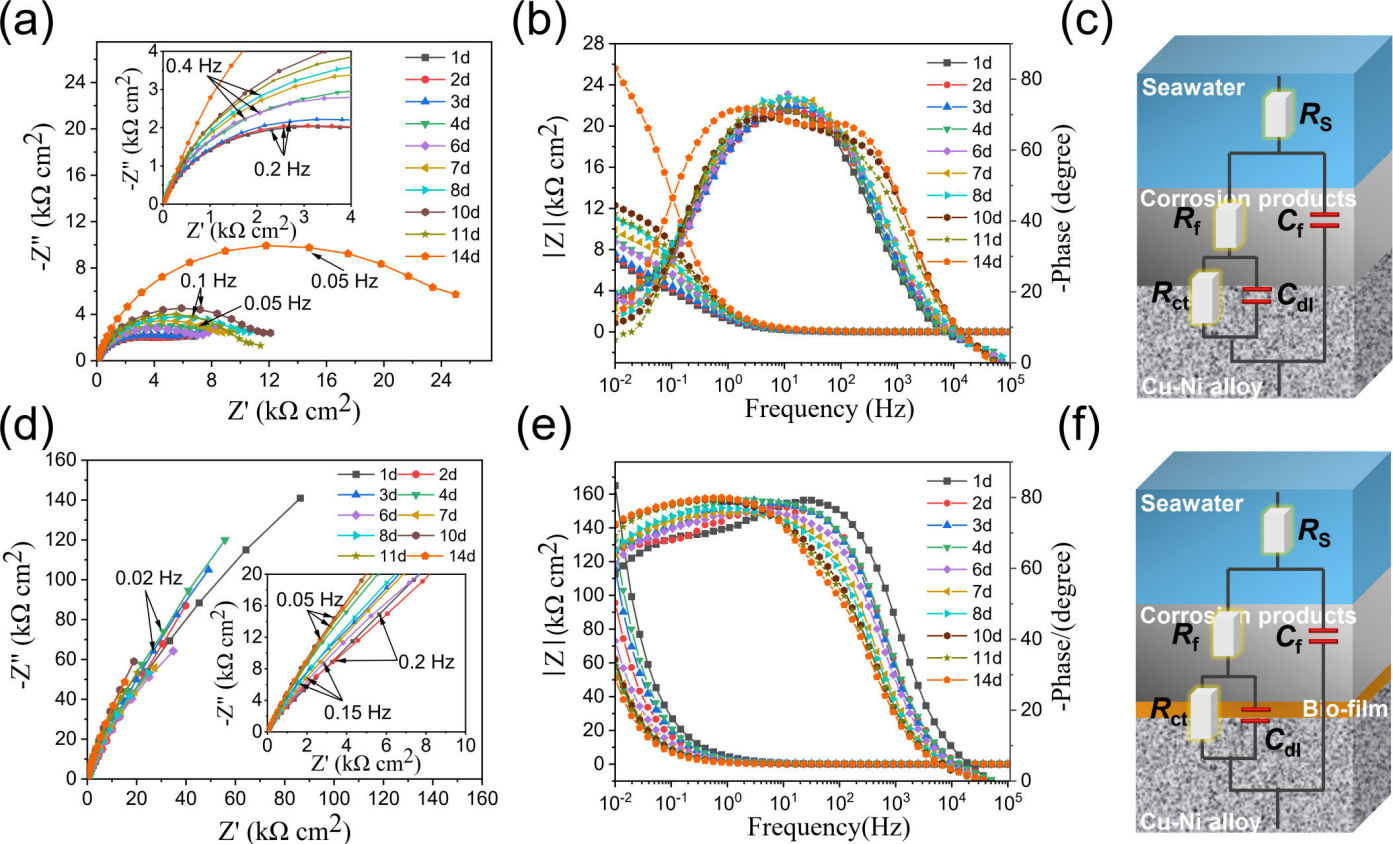
The Nyquist plots, Bode plots, and equivalent circuits of the B30 Cu–Ni alloy during 14 d of immersion in sterile (**a–c**) and *B. subtilis* (**d–f**) systems.

Figure 6 shows the Nyquist and Bode plots of the B30 Cu–Ni alloy in seawater over time. In the sterile system, the diameters of capacitive reactance arcs increased from 1 to 10 d (Fig. 6a), indicating the initial formation of a corrosion product layer that provided some barrier protection. From 10 to 14 d, the diameters first decreased and then increased, reflecting the dynamic and unstable characteristics of the film layer formed in the sterile medium, which was likely due to the continued thickening and increased heterogeneity of the corrosion products. In the Bode plots (Fig. 6b), two characteristic peaks appeared, while the two time constants indicated the formation of a surface film on the alloy surface (22). However, the magnitude of the phase angle initially increased and then decreased, confirming the poor integrity and homogeneity of the surface product film layer. In the test condition containing *B. subtilis*, the diameters of capacitive reactance arcs remained at a higher value throughout the entire immersion period, much greater than that of the sterile system, indicating that the presence of *B. subtilis* inhibited the corrosion of B30 Cu–Ni alloy (Fig. 6d). The phase angle peaks in the *B. subtilis* system were notably broader and shifted toward the lower frequency regions (Fig. 6e), a characteristic behavior attributed to the formation of a dense and integrated biofilm/biomineral composite layer. This surface film served as a physical barrier that effectively impeded the electrochemical reactions at the B30 Cu–Ni alloy interface.

**Fig 7:**
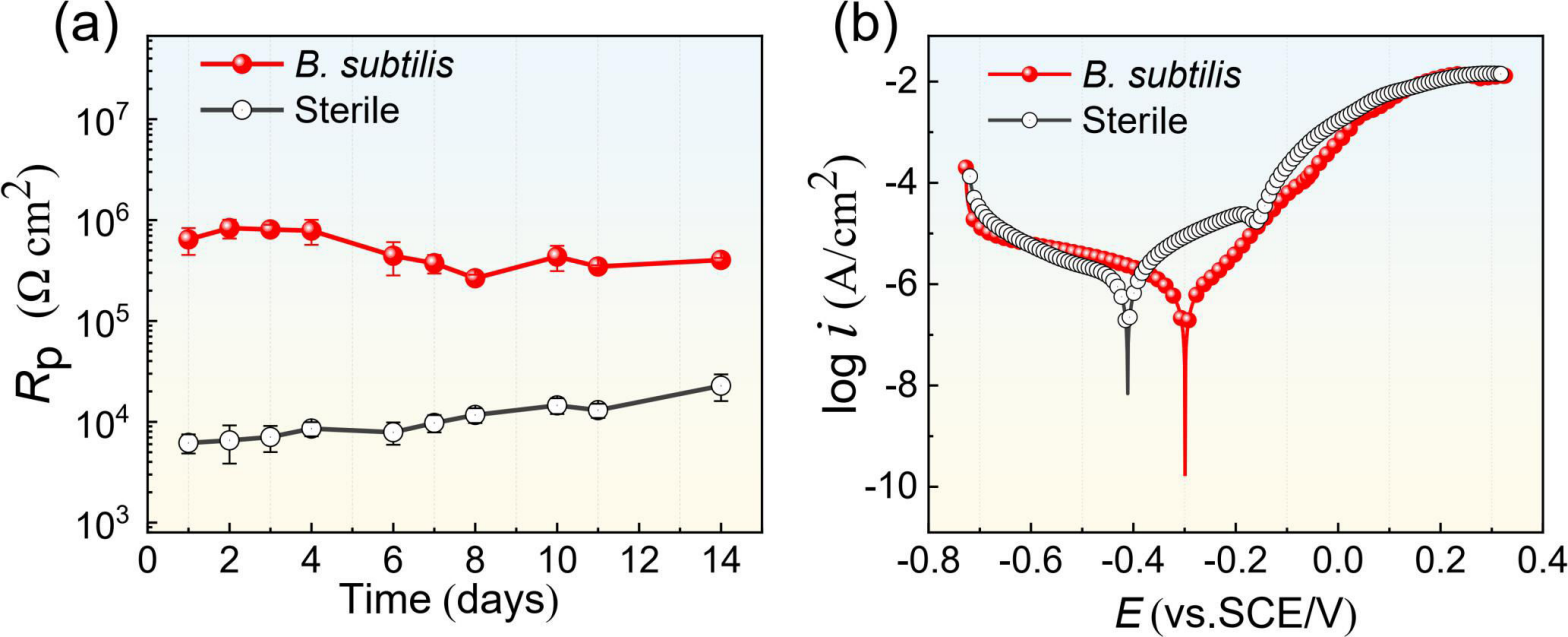
(**a**) Time-dependent *R*_p_ values fitted from EIS data and (**b**) potentiodynamic polarization curves of the B30 Cu–Ni alloy after 14 d of immersion in sterile and *B. subtilis* systems.

To better analyze the corrosion process, the EIS data were fitted with the two-time constants equivalent circuit, as shown in 6c and f. In the equivalent circuits, *R*_s_ represents the solution resistance, CPE_f_ and *R*_f_ represent the constant phase element (including film capacitance *Q*_f_ and dispersion index *n*_f_) and the resistance of the surface film, respectively. CPE_dl_ and *R*_ct_ represent the constant phase element of the electrical double layer (including double layer capacitance *Q*_dl_ and dispersion index *n*_dl_) and the charge transfer resistance, respectively. The fitted impedance parameters are presented in Table S1. The corrosion rate of the B30 Cu–Ni alloy is inversely proportional to the corrosion resistance *R*_p_, that is, the sum of *R*_f_ and *R*_ct_ fitted from the EIS data (23). As shown in Fig. 7a, *R*_p_ remained consistently lower during the 14-d sterile immersion, indicating a corrosion risk of the B30 Cu–Ni alloy in a seawater environment. In contrast, the *R*_p_ values in *B. subtilis* were approximately two orders of magnitude larger than those in the sterile system, suggesting that the corrosion rate was significantly reduced. It further demonstrated that the bacterial biofilm and biomineral layer formed by *B. subtilis* can effectively mitigate B30 Cu–Ni alloy corrosion. Figure 7b shows the potentiodynamic polarization curves of the B30 Cu–Ni alloy after 14 d of immersion in sterile and *B. subtilis* systems, and Table 1 shows the corresponding fitting results, including corrosion potential (*E*_cor_), corrosion current density (*i*_cor_), and anodic and cathodic Tafel slopes (*β*a and *β*c). The fitted *E*_cor_ for the *B. subtilis* system shifted to the more positive direction, and *i*_cor_ decreased by about 2.8 times compared to the sterile system. Furthermore, Tafel slopes reflect the rate-determining steps of cathodic and anodic reactions on the metal surface. Obviously, the value of *β*_c_ in the presence of *B. subtilis* was significantly higher, indicating that the cathodic reaction was retarded to a greater extent than the anodic reaction. These fitting parameters demonstrated that the bacterial biofilm complexed with mineralized products to form a complete film structure, which constructed a uniform protective barrier on the coupon surface, effectively inhibiting the cathodic reaction and corrosion process of the B30 Cu–Ni alloy.

**TABLE 1:** Fitting results of the potentiodynamic polarization curves of B30 Cu–Ni alloy in sterile and *B. subtilis* systems

	*E*_cor_ (V)	*i*_cor_ (A/cm^2^)	*β*_a_ (V/dec)	*β*_c_ (V/dec)
Sterile	−0.41 ± 0.01	(2.11 ± 0.10) × 10^−6^	0.124 ± 0.008	0.201 ± 0.018
*B. subtilis*	−0.32 ± 0.03	(5.85 ± 0.08) × 10^−7^	0.110 ± 0.005	0.414 ± 0.006

### Biomass regulation

**Fig 8:**
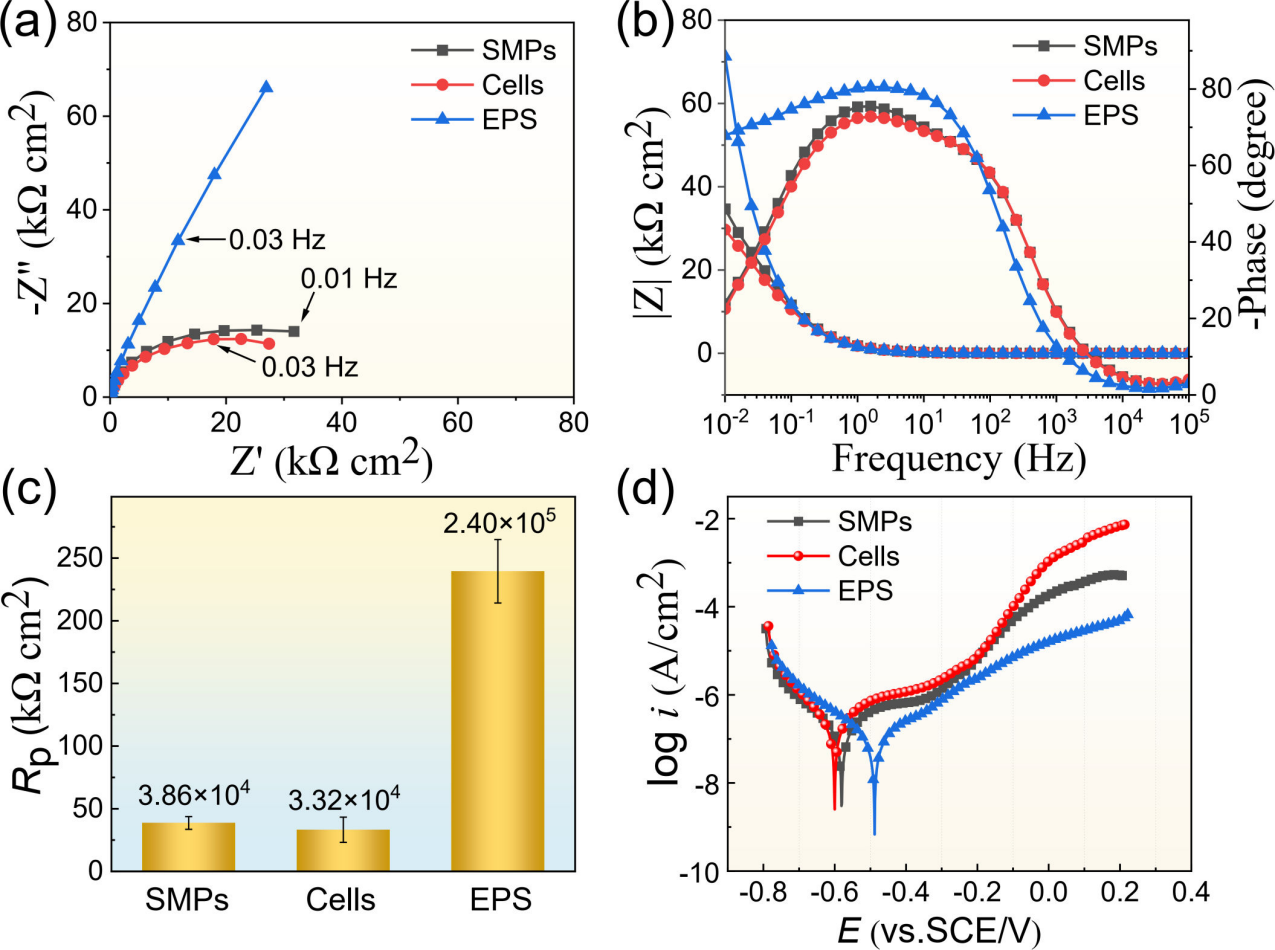
The Nyquist plots (**a**), Bode plots (**b**), *R*_p_ values (**c**), and potentiodynamic polarization curves (**d**) of the B30 Cu–Ni alloy after 14 d of immersion in different *B. subtilis* biomass systems.

To elucidate the corrosion inhibition mechanism of *B. subtilis*, bacterial cells, EPS, and SMPs were isolated from the bacterial culture. The EIS data of B30 Cu–Ni alloy after immersion in *B. subtilis* biomass for different times are shown in Fig. S5. It can be seen that the diameters of Nyquist plots in the EPS were significantly larger than those in the cells and SMPs systems, exhibiting excellent corrosion resistance due to the adsorption of organic matter in EPS onto the alloy surface (24). During the initial stage (1–7 d), the impedance arc diameters of the cells were larger than that of the SMPs, indicating that *B. subtilis* bare cells exerted a certain inhibitory effect on B30 Cu–Ni alloy corrosion. However, as the immersion time extended to 14 d (Fig. 8a and b), the impedance arc diameters of SMPs showed an upward trend, the peak phase angle shifted toward a low-frequency region, and both the maximum peak phase angle and the modulus value increased. This trend can be attributed to the fact that organic acids in SMPs accelerated initial corrosion, while the corrosion products formed on the metal surface subsequently exerted a protective effect, thereby reducing the corrosion rate. Consistently, the *R*_p_ value was higher in the presence of the EPS, representing a lower corrosion rate. Figure 8d shows the potentiodynamic polarization curves of the B30 Cu–Ni alloy after immersion in *B. subtilis* cells, EPS, and SMPs medium for 14 d. The corresponding fitting results are shown in Fig. S3. Compared to the cell and SMPs groups, the polarization curves in the medium containing EPS showed a significant rightward shift, and both the anodic and cathodic reactions were significantly suppressed, resulting in a decrease in the corrosion current, demonstrating that EPS provided more effective protection for the B30 Cu–Ni alloy.

**Fig 9:**
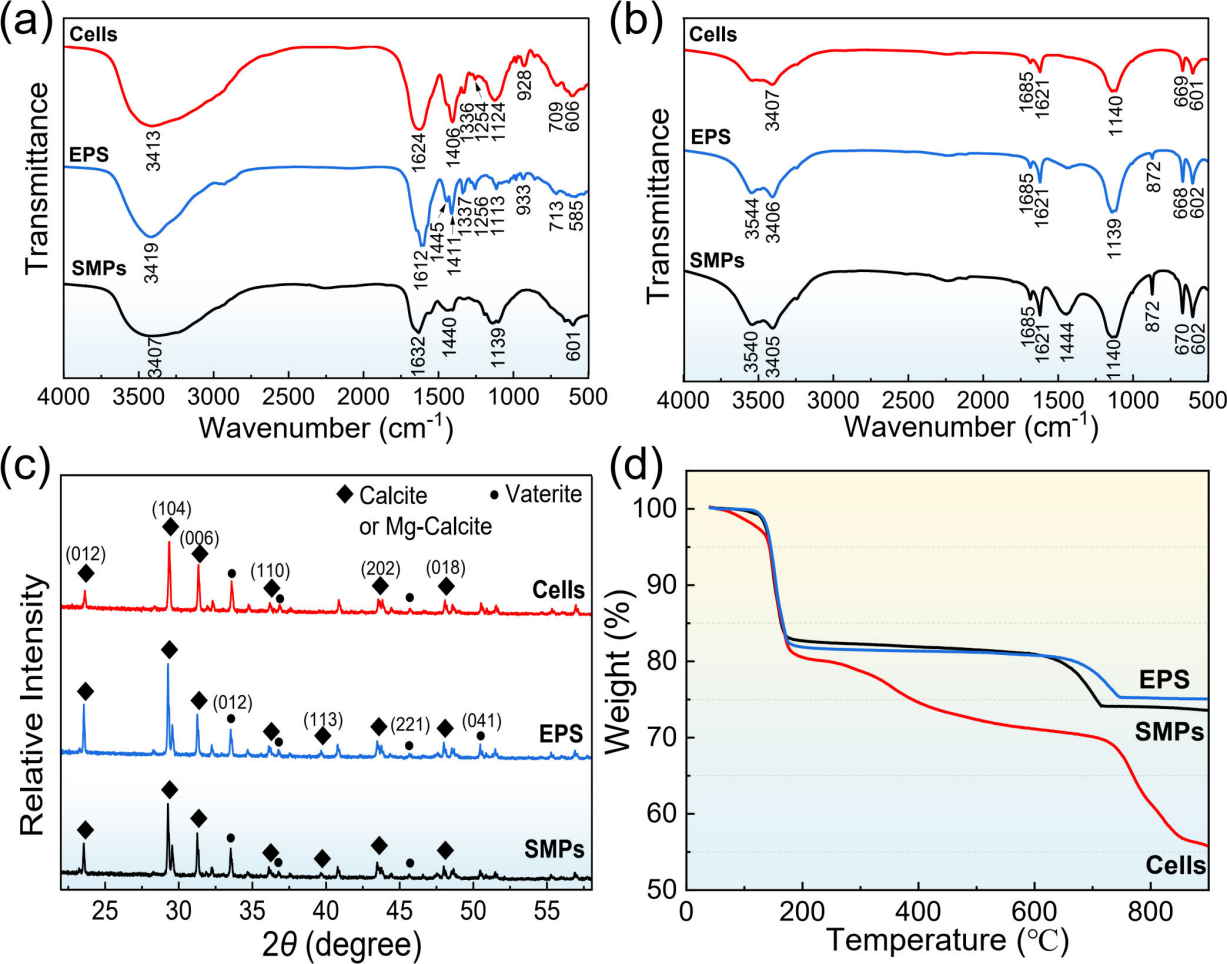
(**a**) FT-IR spectra of different biomass of *B. subtilis* and (**b**) corresponding biomass mineralized products; (**c**) XRD patterns and (**d**) TGA curves of mineralized products in the presence of different biomass components.

Figure 9a shows the FT-IR spectral analysis of different biomass, in which 1,632 cm^–1^, 1,612 cm^–1^, and 1,624 cm^–1^ were the characteristic peaks of C=O stretching vibration in carboxyl groups of proteins, and the bands near 1,440 cm^–1^ and 1,139 cm^–1^ were related to the N–H in amino groups and C–O in phenolic groups. The broad absorption band near 3,407 cm^–1^ was formed by the stretching vibration of O–H bond in hydroxyl functional groups of polysaccharides and proteins. In contrast, the O–H in-plane bending vibration (near 1,337 cm^−1^) and P = O bond (near 1,256 cm^−1^) were present only in cells and EPS, indicating that phosphorus compounds were caused by the presence of phospholipid bilayer and nucleic acid substances in cells and EPS. Figure 9b shows the FT-IR analysis results of mineralized products after adding corresponding biomass components for 72 h. It can be seen that all experimental groups had peaks near 1,685 cm^−1^ and 3,540 cm^−1^, which were attributed to C = O bond stretching vibration as well as intra- and intermolecular hydrogen bonding interaction of carboxylic acids (25). In addition, the EPS and SMP groups showed the calcite characteristic absorption peaks at 872 cm^−1^ and 1,444 cm^−1^ of the outer plane bending vibration (*v*_4_) in carbonates (26). In the XRD pattern (Fig. 9c), the main characteristic peaks were consistent, and the mineralization products were dominated by calcite, with a small amount of vaterite (27). It was noted that the 2*θ* angle of calcite was slightly shifted relative to the standard calcite diffraction peak of PDF#05-0586, which may be due to lattice contraction caused by the presence of Mg in the calcite crystal (28). Figure 9d presents the TGA curves of the mineralization products formed by different biomass. There were four weight-loss steps for cell-induced products: the thermal weight-loss of the first step from room temperature to 130℃ was 2.96%, which was related to the water adsorption on the crystal surface. The second weight-loss step (16.51%) occurred in the range of 130°C–200°C, implying the decomposition of organic matter in minerals. The thermal weight loss in 200°C–650°C was attributed to the decomposition of Mg carbonate, which was about 9.84%. The last weight-loss step occurred between 650°C and 850°C, corresponding to the thermal decomposition of CaCO_3_ into CaO and CO_2_. For the EPS and SMP groups, no new weight-loss steps were observed in TGA curves, but the overall weight loss was significantly reduced, reflecting the advantageous thermal stability of mineralized products.

**Fig 10:**
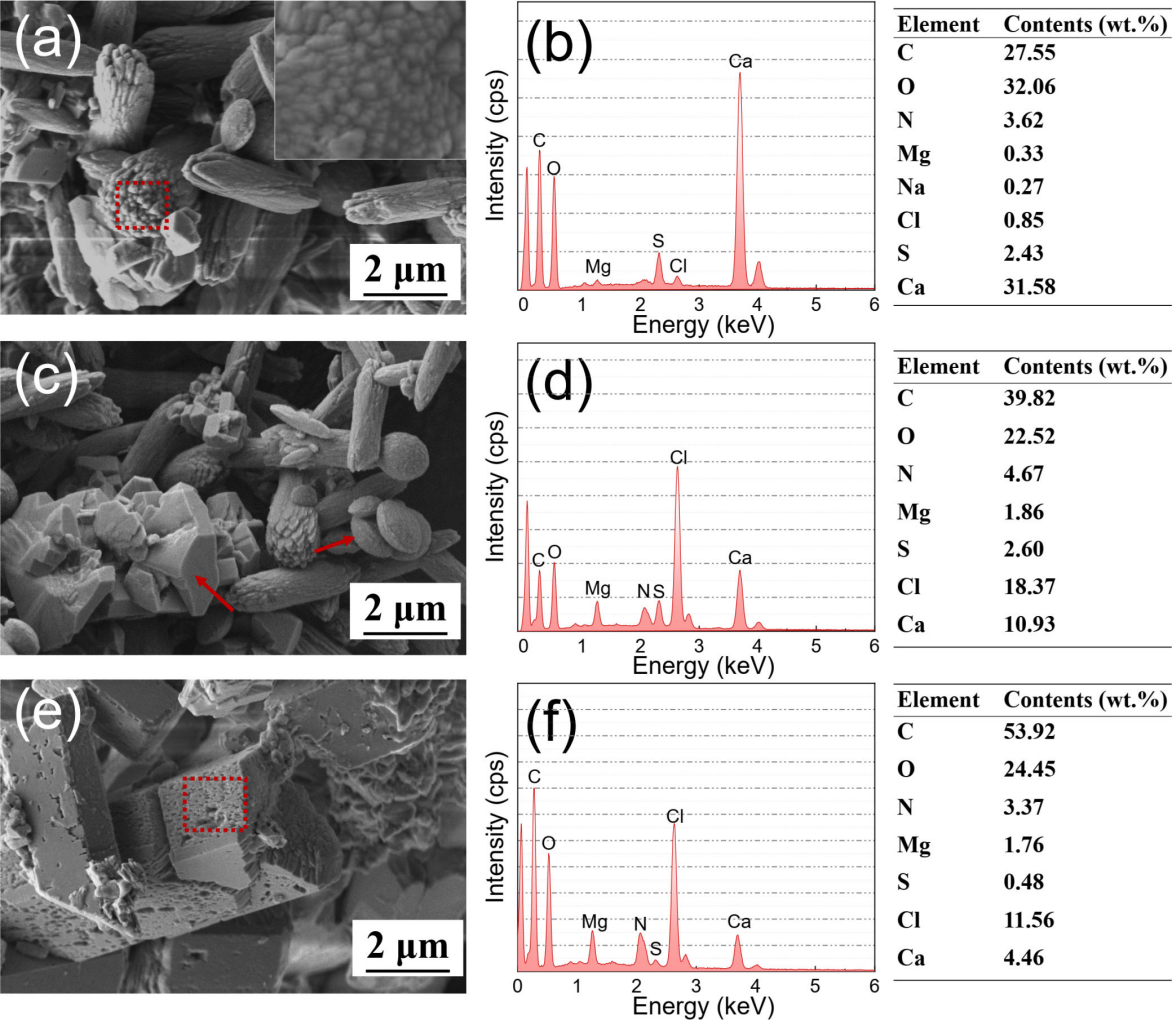
SEM images and EDS analysis of mineralized products in the presence of different biomass components and the corresponding element contents: (**a and b**) cells; (**c and d**) EPS and (**e and f**) SMPs.

The SEM images and EDS analysis of the mineralized products with different biomass components for 72 h are shown in Fig. 10. The crystallization products of the cells not only exhibited elongated characteristics, but also some trigonal rhombohedral calcite structures when viewed close up (Fig. 10a, insert) (29). EDS analysis shows that the mineralization products were mainly composed of C, O, and Ca (Fig. 10b). In the case of EPS, ellipsoidal vaterite and irregular rhombohedral calcite coexist (Fig. 10c), with a pronounced Mg elemental peak (Fig. 10d), indicating that EPS contributed to the complexation of Ca^2+^ and Mg^2+^ ions to form Mg-calcite (30). In the SMPs group, blocky Ca-Mg carbonates with rough surfaces have been observed (Fig. 10e). EDS analysis revealed the presence of microbiologically essential elements, such as C, N, and S, suggesting that *B. subtilis* and its metabolites were involved in the generation and transformation of Ca-Mg carbonates.

**Fig 11:**
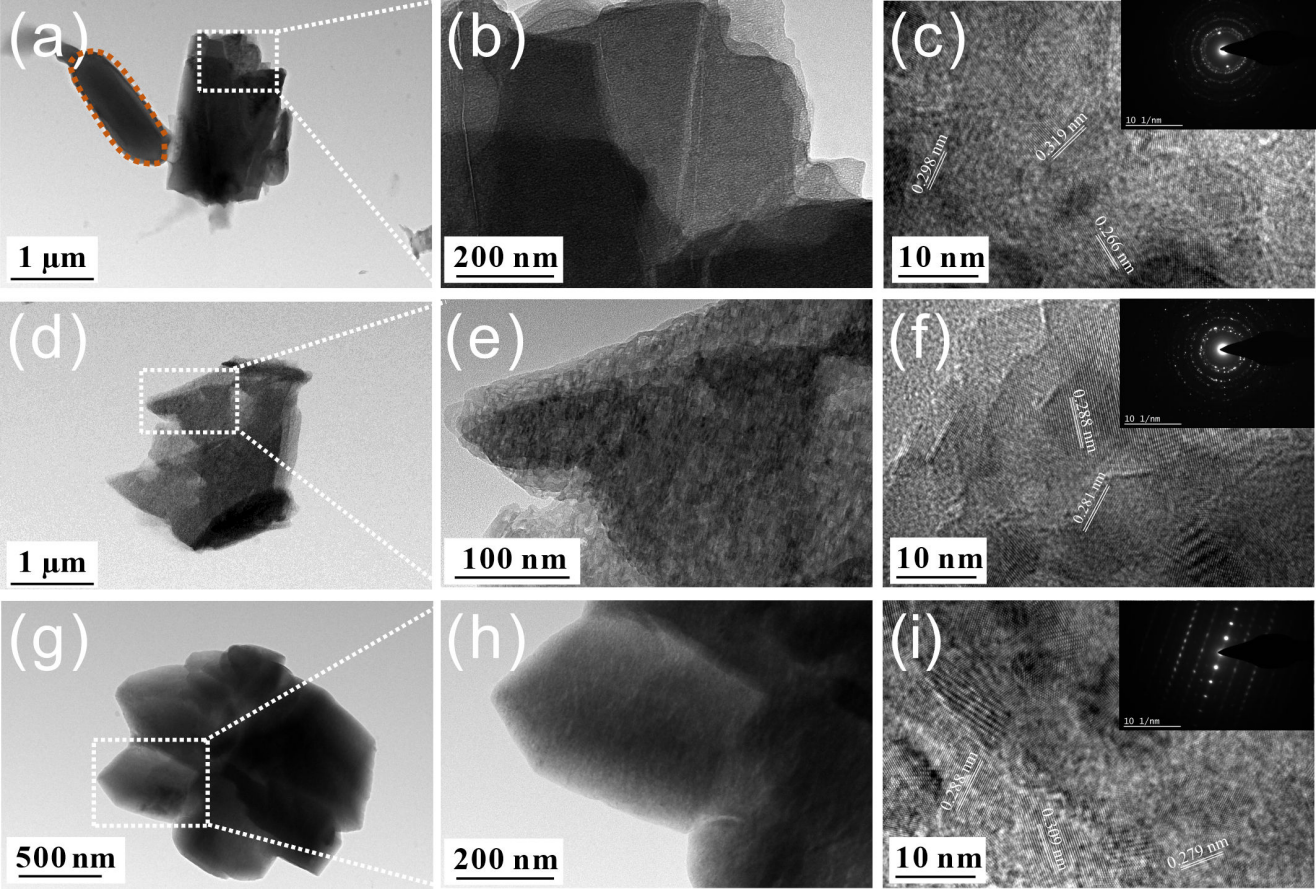
TEM images and SAED patterns of mineralized products formed by different biomass components of *B. subtilis*: (**a–c**) cells; (**d–f**) EPS and (**g–i**) SMPs.

The morphology and internal structure of the biominerals under the regulation of different biomass of *B. subtilis* were characterized by TEM, as shown in Fig. 11. When induced by bacterial cells, the precipitates consisted of elongated crystalline particles (~2 µm), exhibiting heterogeneous internal contrast (Fig. 11a). This contrast variation likely originated from thickness effects due to the overlapping of multiple crystalline domains (31). Interestingly, the observed rod-shaped cellular structure adjacent to the minerals confirmed the essential templating role of bacterial cells in directing mineralization. HRTEM (Fig. 11c) revealed lattice spacings of 0.266 nm, 0.298 nm, and 0.319 nm, corresponding to calcite planes (32). The SAED pattern (Fig. 11c, inset), showing diffraction spots and diffuse rings, indicated a composite of nanocrystalline and amorphous phases (33). In the EPS-induced system, the precipitates consisted of sub-micrometer, polycrystalline aggregates (Fig. 11d). The measured lattice spacing of ~0.28 nm and the corresponding SAED pattern (Fig. 11f) confirmed the phase as Mg-rich calcite (34). The incorporation of Mg²^+^ into the calcite structure likely enhanced the compactness and continuity of the surface layer, thereby improving its barrier protective performance and contributing to the enhanced corrosion resistance observed in electrochemical measurements (Fig. 8). In the presence of SMPs, the uniform contrast in TEM (Fig. 11g and h) and the spot-like SAED pattern (Fig. 11i) revealed the single-crystalline nature of these precipitates (35). Such highly crystalline particles can form a less permeable barrier layer, effectively blocking the diffusion of corrosive substances, thereby increasing electrical impedance and reducing corrosion current.

## DISCUSSION

In this study, the B30 Cu–Ni alloy exhibited high corrosion resistance in the presence of *B. subtilis*, with the maximum pit depth drastically reduced from 44.74 to 18.54 µm (Fig. 5). Results show that corrosion inhibition was attributed to the biofilm and Ca-Mg carbonates (i.e., the biomineral layer) formed on the alloy surface (Fig. 1 to 4). The barrier protection conferred by the biomineral layer suppressed the cathodic oxygen reduction reaction and limited the transport of corrosive species, resulting in a low corrosion current of (5.85 ± 0.08) × 10^−7^ A/cm² (Table 1). This explains why the *R*_p_ values in the *B. subtilis* group were much higher than those in the control group (Fig. 7a), indicating the superior anti-corrosion performance imparted by the biomineral layer. However, the distinct biomineral characteristics induced by different biomass components directly govern their corrosion inhibition efficacy and durability. The Mg-calcite formed in the EPS system demonstrated superior chemical stability compared to the bare cells system (Fig. 9d). Furthermore, the compact polycrystalline structure of EPS-induced minerals (Fig. 11f), characterized by tightly packed nanocrystals with clearly defined lattice fringes, created a compact protective layer against corrosive species penetration in seawater. This microstructure corresponded to the higher impedance values observed in EIS measurements during 14 d of immersion (Fig. S5), indicating that the EPS-induced layer exhibited more durable anti-corrosion properties. In comparison, the single-crystalline calcite generated in the SMPs system, while well-crystallized, may be more susceptible to localized dissolution at the crystal boundaries (36), resulting in a lower protection efficiency compared to the EPS system (Fig. 8).

**Fig 12:**
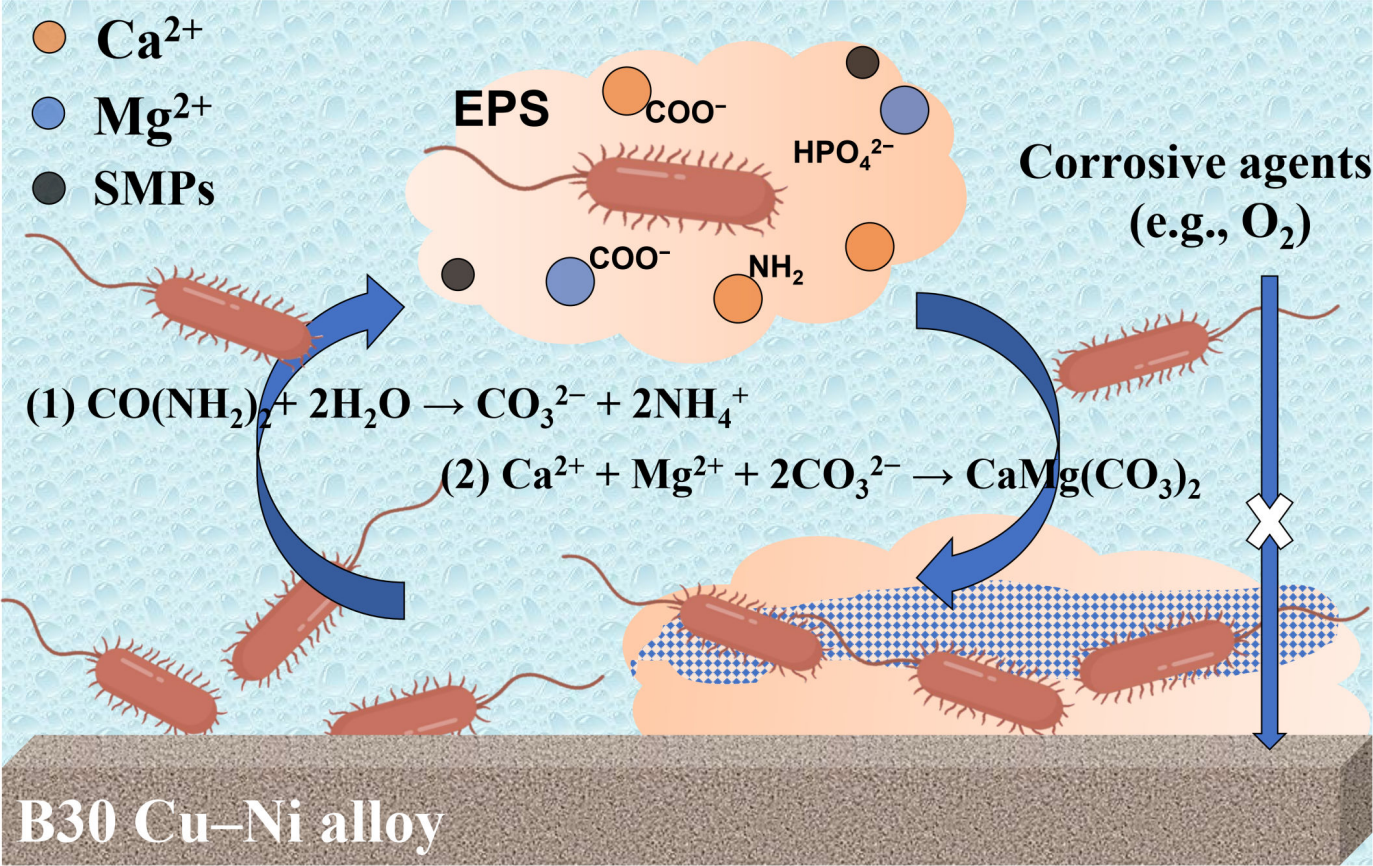
Schematic illustration of the corrosion inhibition mechanism of *B. subtilis* on the surface of B30 Cu–Ni alloy.


In general, the corrosion inhibition mechanism of *B. subtilis* on B30 Cu–Ni alloy is illustrated in Fig. 12, mainly reflected in the biomass templating and nucleation, the mineral barrier formation, and the electrochemical corrosion inhibition on the alloy surface. The process initiated with the adsorption of bacterial biomass onto the alloy surface. Both bacterial cells and their secreted EPS are rich in negatively charged functional groups such as carboxyl, phosphoryl, and amino. These groups can complex with Ca²^+^ and Mg²^+^ ions in seawater, forming a local ion-enriched microenvironment and serving as effective nucleation sites and structural templates for mineral precipitation (37). FT-IR analysis confirmed the complexation of these functional groups with metal ions (Fig. 9b), and TEM provided direct evidence of bacterial cells acting as nucleation templates (Fig. 11a). Subsequently, the metabolic activities of *B. subtilis* (such as urea hydrolysis) would regulate the local chemistry at the alloy/solution interface, as shown in equations (1) to (3)-3 (38).

(1) $$CO(NH_{2}{)}_{2}+2H_{2}O\to CO_{3}{\;}^{2-}+2NH_{4}{\;}^{+}$$

(2) $$Ca^{2+}+CO_{3}{\;}^{2-}\to CaCO_{3}$$

    or


(3) $$Ca^{2+}+Mg^{2+}+2CO_{3}{\;}^{2-}\to CaMg(CO_{3}{)}_{2}$$

This metabolism increases the local pH and the concentration of CO_3_²⁻ ions, thereby promoting the supersaturation for Ca²^+^ and Mg²^+^ carbonates (39). The uniform coverage and elemental composition (Ca, Mg, C, and O) of the biomineral layer were observed by SEM and elemental mapping (Fig. 2d and 3), while its crystalline phases (calcite, Mg-calcite, and vaterite) were identified by XRD and TEM (Fig. 9c and 11). Finally, the *B. subtilis* biofilm and mineral layer acted as a physical barrier on the alloy surface, significantly impeding the mass transfer of corrosive agents, such as blocking the transport of dissolved oxygen to the cathode sites on the surface of B30 Cu–Ni alloy (increasing the cathodic Tafel slope *β*c). The EIS results showed a drastic increase in the corrosion resistance (Fig. 6 and 7a), while the potentiodynamic polarization curve recorded a low corrosion current (Fig. 7b), demonstrating the excellent corrosion inhibition performance of *B. subtilis*.

### Conclusions

In this work, it was demonstrated that *B. subtilis* and its biomass components effectively inhibited the corrosion of B30 Cu–Ni alloy in seawater. The corrosion protection was achieved through the following pathways: (i) biomass templating, where bacterial cells and EPS adsorbed to the alloy surface and provided nucleation sites through the negatively charged functional groups (e.g., carboxyl, phosphoryl, and amino) that complexed with Ca²^+^ and Mg²^+^ ions in seawater; (ii) mineral barrier formation, where the metabolic activity of *B. subtilis* regulated the local microenvironment, promoting the growth of a continuous and adherent composite layer of Ca-Mg carbonates; and (iii) electrochemical corrosion inhibition, where the significant increase in cathodic Tafel slope *β*_c_, coupled with the reduction in corrosion current, indicated that the biomineral layer acted as a physical barrier, hindering the diffusion of corrosive agents (e.g., O_2_) to the alloy surface, thereby suppressing the cathodic reaction and the overall corrosion process. This study not only elucidates the biomass-regulated corrosion inhibition mechanism of *B. subtilis* but also establishes a scientific basis for developing targeted and sustainable anti-corrosion strategies based on microbial principles.

## Data Availability

The relevant data are available from the corresponding authors upon reasonable request.

## SUPPLEMENTAL MATERIAL

Supplemental material aem.02286-25-s0001.docx Figures S1 to S5; Tables S1 to S3.

ASM does not own the copyrights to Supplemental Material that may be linked to, or accessed through, an article. The authors have granted ASM a non-exclusive, world-wide license to publish the Supplemental Material files. Please contact the corresponding author directly for reuse.
