# Supplementary material for: Influence of Bacillus subtilis on the corrosion resistance of B30 copper–nickel alloy and the biomass-regulated mineralization mechanism
Source: Appl Environ Microbiol. 2025 Dec 10;92(1):e02286-25. doi: 10.1128/aem.02286-25 (PMC12838447; doi:10.1128/aem.02286-25)
Supplement: Supplemental material — Figures S1 to S5; Tables S1 to S3. [file aem.02286-25-s0001.docx]

**Influence of *Bacillus subtilis* on the corrosion resistance of B30 copper-nickel alloy and the biomass-regulated mineralization mechanism**

**Meiying Lv ^a *^, Lixian Chen ^a^, Xingyi Tang** **^a^, Ruoxi Huang ^a^, Min Du ^b *^, Xiyun Zhang ^b^, Xingchuan Zhao ^a^, Yan Li ^a^ and Yongxu Du ^a^**

^a^ School of Materials Science and Engineering, Liaocheng University, Liaocheng 252059, China

^b^ Key Laboratory of Marine Chemistry Theory and Technology, Ministry of Education, College of Chemistry and Chemical Engineering, Ocean University of China, Qingdao 266100, China

^*^Corresponding author.

E-mail: [lvmeiying@lcu.edu.cn](mailto:lvmeiying@lcu.edu.cn); ssdm99@ouc.edu.cn;


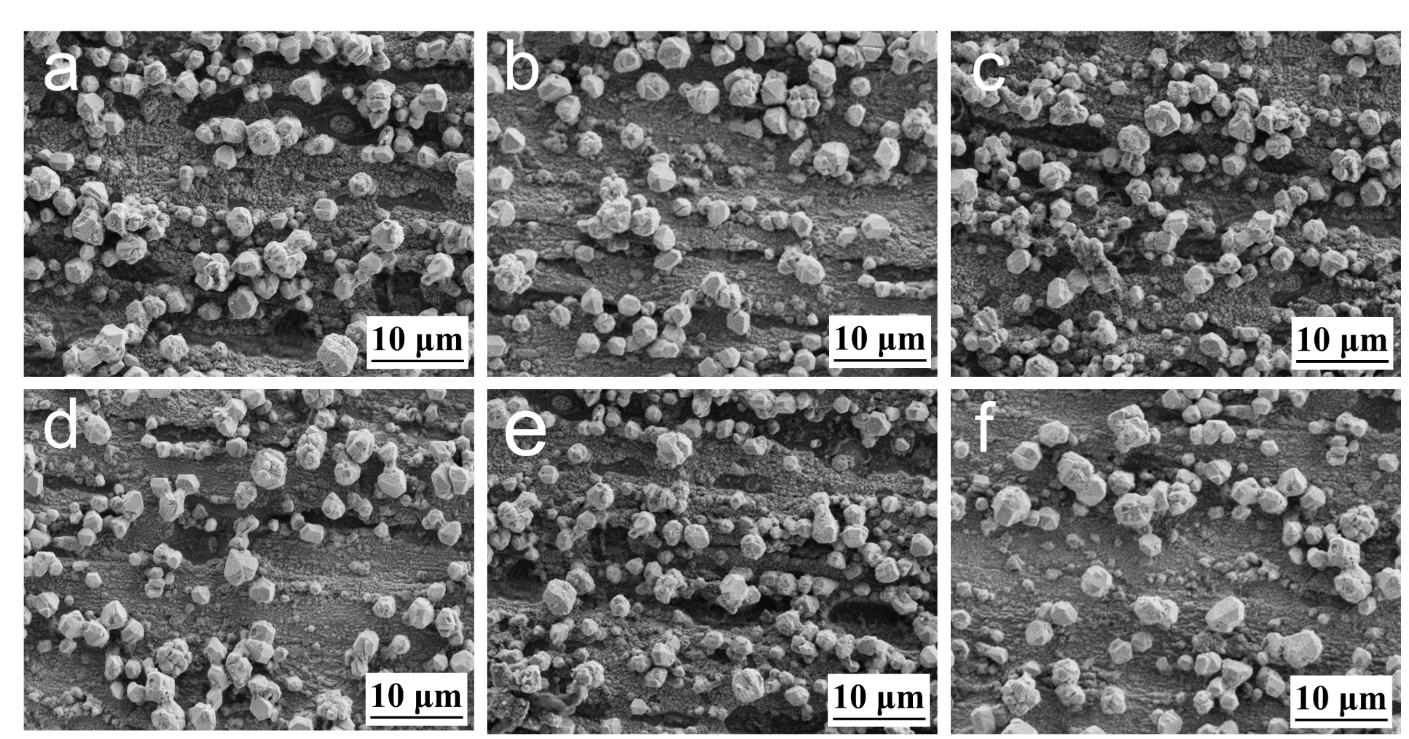


**Fig. S1.** SEM images of different regions on the B30 Cu–Ni alloy after immersion in the *B. subtilis* medium for 14 d.


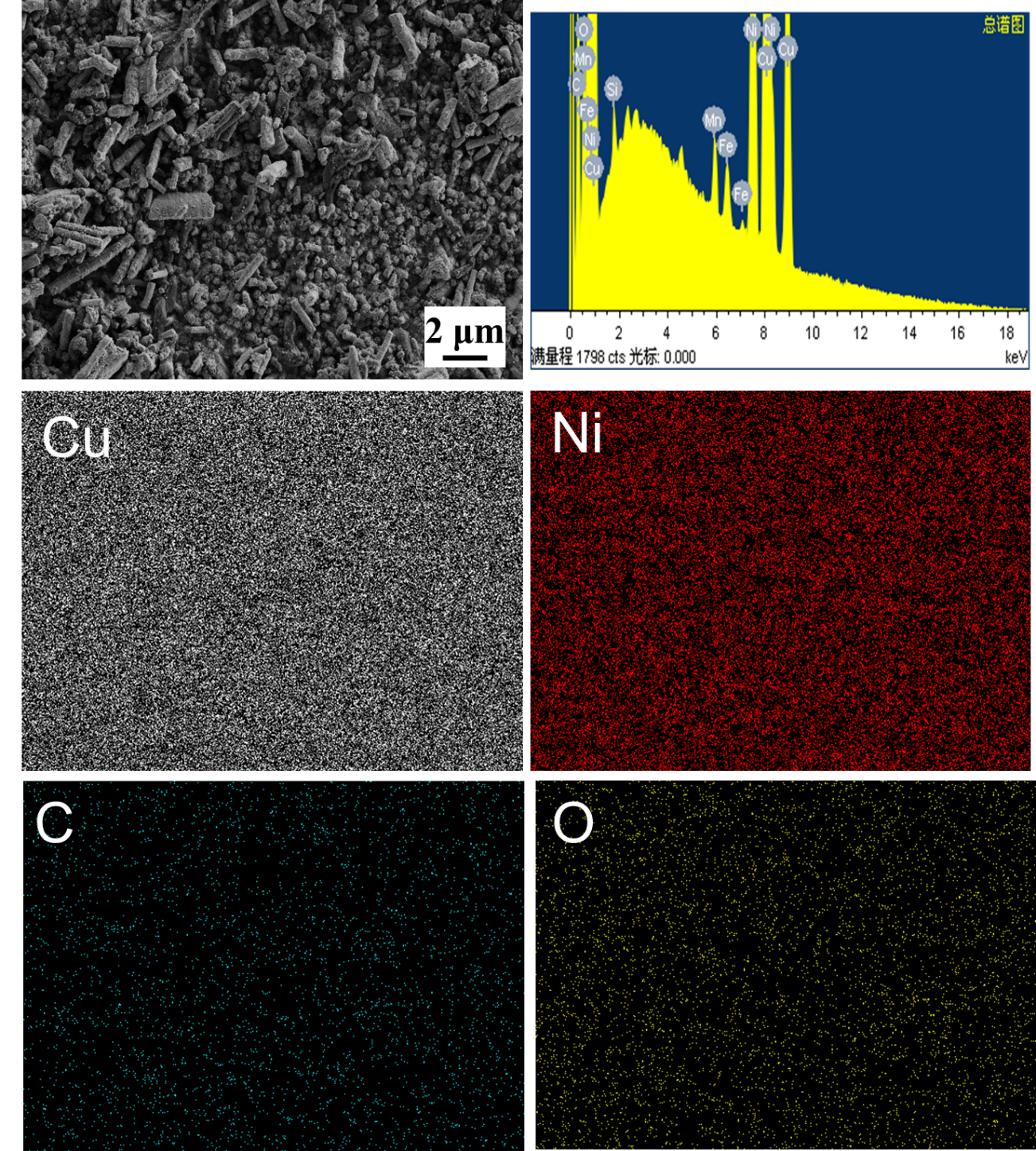


**Fig. S2.** EDS and elemental mapping images of Cu, Ni, C and O elements for B30 Cu–Ni alloy after immersion for 14 d in the sterile medium.


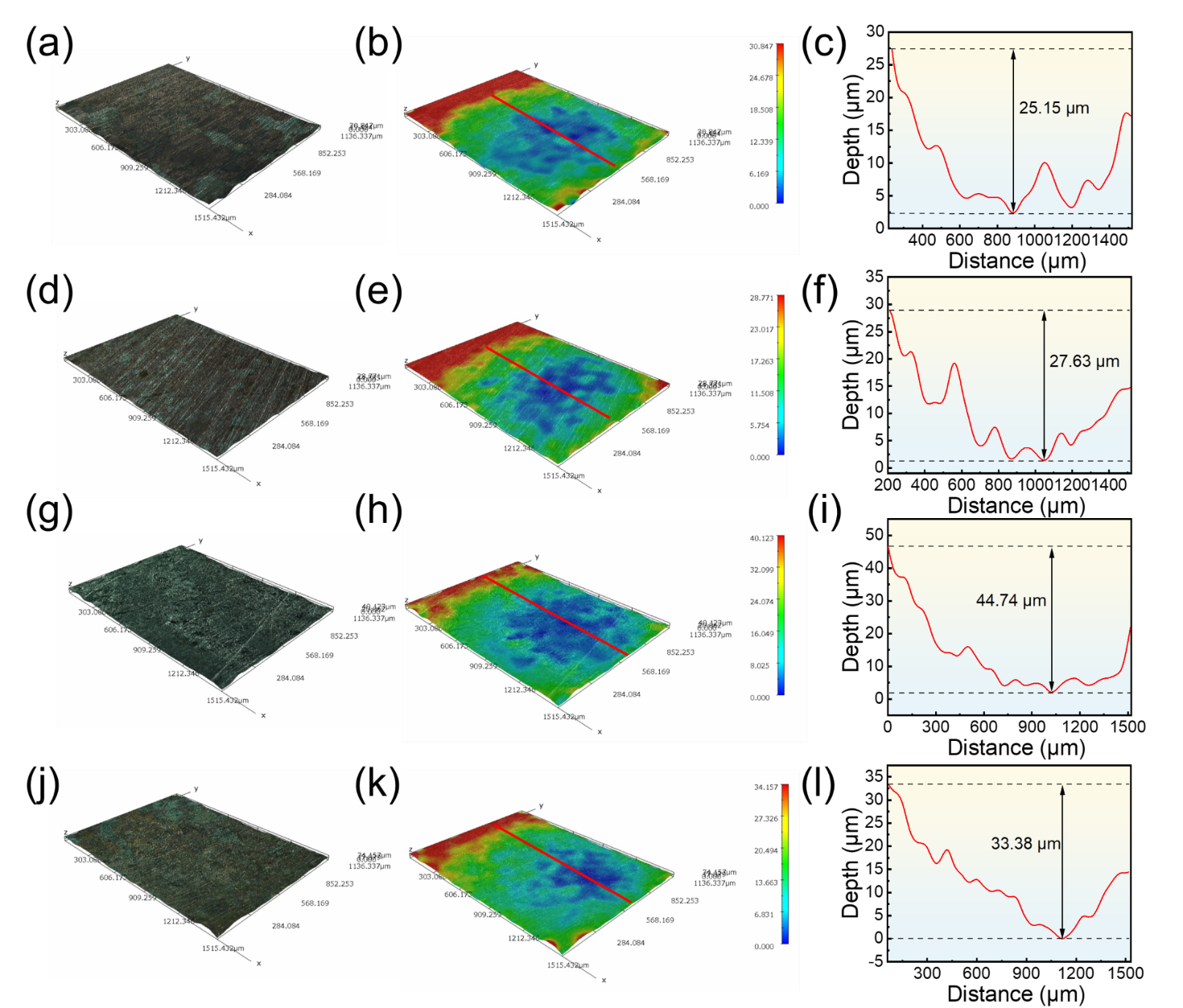


**Fig. S3.** Corrosion morphology (a, d, g, j), 3D profile (b, e, h, k), and pit depth (c, f, i, l) at red line of the B30 Cu–Ni alloy after 14 d of immersion in the sterile system.


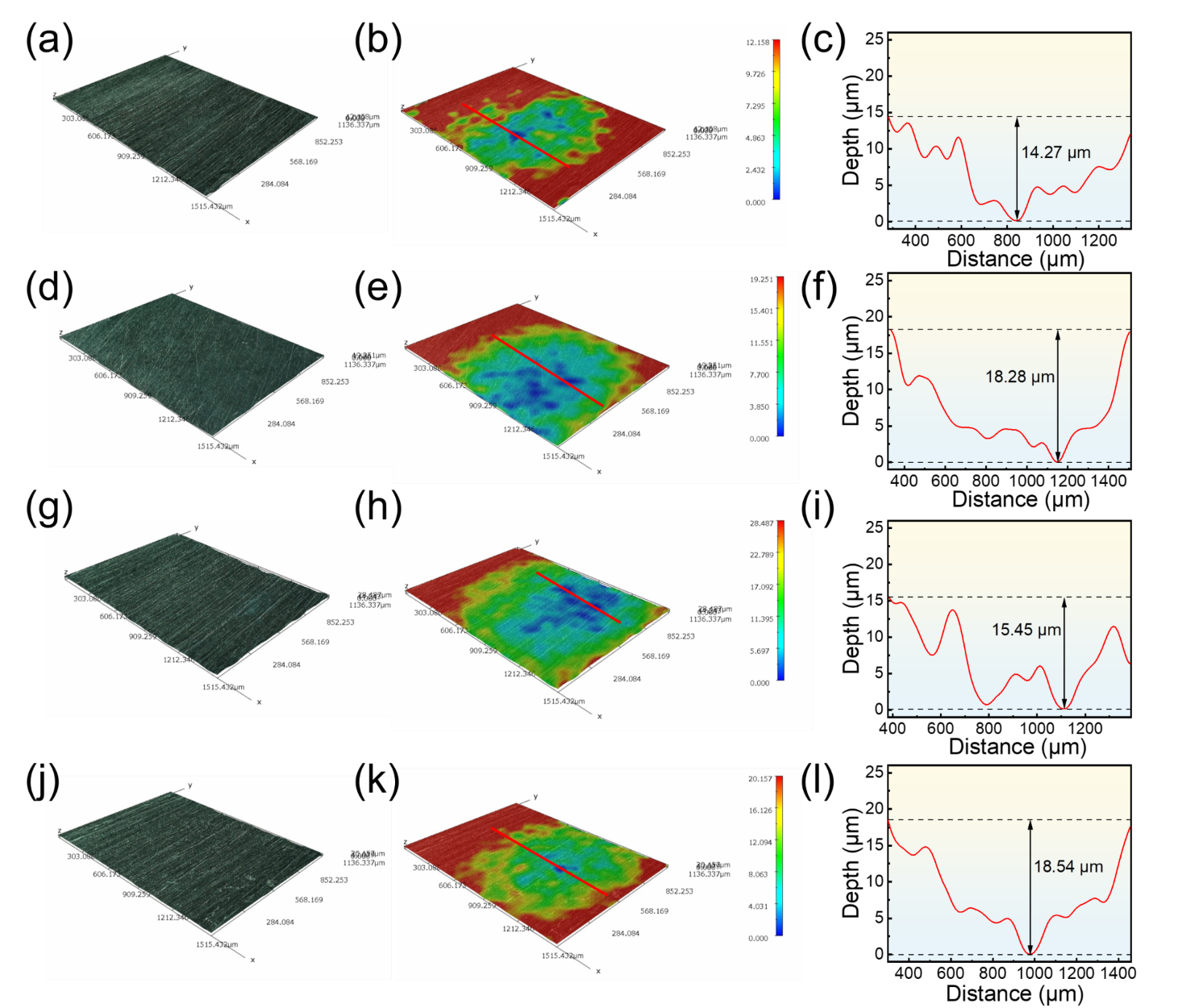


**Fig. S4.** Corrosion morphology (a, d, g, j), 3D profile (b, e, h, k), and pit depth (c, f, i, l) at red line of the B30 Cu–Ni alloy after 14 d of immersion in the *B. subtilis* system.

**Table S1** EIS fitting parameters of the B30 Cu–Ni alloy immersed in sterile and *B. subtilis* systems.

|  | *t*  (d) | *R*_s_  (Ω·cm^2^) | *R*_ct_  (Ω·cm^2^) | *Q*_dl_×10^−5^  (F·cm^−2^) | *n*_dl_ | *R*_f_  (Ω·cm^2^) | *Q*_f_×10^−4^  (F·cm^−2^) | *n*_f_ |
| --- | --- | --- | --- | --- | --- | --- | --- | --- |
| Sterile | 1 | 7.24±0.55 | (6.12±0.37)×10^3^ | 2.72±0.32 | 0.74±0.06 | 57.54±16.50 | 1.58±0.29 | 0.78±0.06 |
|  | 2 | 6.51±0.78 | (6.47±0.63)×10^3^ | 3.11±0.27 | 0.75±0.07 | 70.68±14.62 | 1.40±0.23 | 0.80±0.03 |
|  | 3 | 6.81±0.34 | (6.97±0.55)×10^3^ | 3.23±0.69 | 0.77±0.10 | 89.45±20.57 | 1.38±0.17 | 0.85±0.07 |
|  | 4 | 7.42±0.48 | (8.40±0.08)×10^3^ | 3.54±0.25 | 0.77±0.13 | (1.54±0.55)×10^2^ | 1.15±0.22 | 0.86±0.12 |
|  | 6 | 6.84±0.25 | (7.72±0.40)×10^3^ | 3.16±0.39 | 0.78±0.09 | (1.68±0.30)×10^2^ | 0.85±0.32 | 0.84±0.05 |
|  | 7 | 6.87±0.69 | (9.57±0.22)×10^3^ | 3.41±0.65 | 0.75±0.11 | (1.78±0.32)×10^2^ | 0.80±0.26 | 0.85±0.03 |
|  | 8 | 6.90±0.57 | (1.15±0.09)×10^4^ | 3.62±0.66 | 0.76±0.09 | (1.90±0.26)×10^2^ | 0.77±0.10 | 0.83±0.11 |
|  | 10 | 6.41±0.34 | (1.43±0.29)×10^4^ | 4.52±0.54 | 0.77±0.05 | (2.41±0.43)×10^2^ | 0.55±0.15 | 0.87±0.05 |
|  | 11 | 6.59±0.57 | (1.28±0.16)×10^4^ | 4.05±0.15 | 0.76±0.07 | (2.15±0.40)×10^2^ | 0.61±0.23 | 0.89±0.06 |
|  | 14 | 6.56±0.63 | (2.23±0.54)×10^4^ | 3.32±0.67 | 0.80±0.05 | (4.97±0.75)×10^2^ | 0.57±0.19 | 0.87±0.07 |
| *B. subtilis* | 1 | 8.04±0.52 | (6.27±0.63)×10^5^ | 2.30±0.15 | 0.75±0.10 | (1.44±0.21)×10^4^ | 0.39±0.18 | 0.90±0.07 |
|  | 2 | 8.40±0.71 | (8.02±0.54)×10^5^ | 3.57±0.54 | 0.73±0.08 | (3.12±0.46)×10^4^ | 0.71±0.11 | 0.88±0.06 |
|  | 3 | 8.15±0.25 | (7.14±0.38)×10^5^ | 3.18±0.67 | 0.78±0.11 | (9.13±0.75)×10^4^ | 0.69±0.05 | 0.88±0.03 |
|  | 4 | 8.66±0.33 | (5.72±0.65)×10^5^ | 3.70±0.32 | 0.82±0.07 | (2.15±0.24)×10^5^ | 0.67±0.08 | 0.87±0.07 |
|  | 6 | 8.04±0.51 | (4.03±0.73)×10^5^ | 2.64±0.21 | 0.73±0.12 | (4.02±0.72)×10^4^ | 1.22±0.35 | 0.88±0.05 |
|  | 7 | 8.13±0.46 | (1.69±0.52)×10^5^ | 4.54±0.56 | 0.92±0.06 | (2.04±0.67)×10^5^ | 1.35±0.19 | 0.85±0.05 |
|  | 8 | 7.88±0.78 | (2.63±0.09)×10^5^ | 4.27±0.69 | 0.93±0.04 | (1.55±0.54)×10^3^ | 1.74±0.21 | 0.85±0.03 |
|  | 10 | 8.26±0.34 | (4.34±0.61)×10^5^ | 2.78±0.62 | 0.95±0.02 | (2.21±0.31)×10^2^ | 1.59±0.44 | 0.90±0.08 |
|  | 11 | 7.79±0.75 | (3.46±0.37)×10^5^ | 3.07±0.34 | 0.96±0.03 | (1.83±0.47)×10^2^ | 1.63±0.32 | 0.86±0.10 |
|  | 14 | 7.85±0.69 | (4.02±0.15)×10^5^ | 3.88±0.35 | 0.97±0.02 | (1.32±0.24)×10^2^ | 1.75±0.14 | 0.88±0.08 |

**
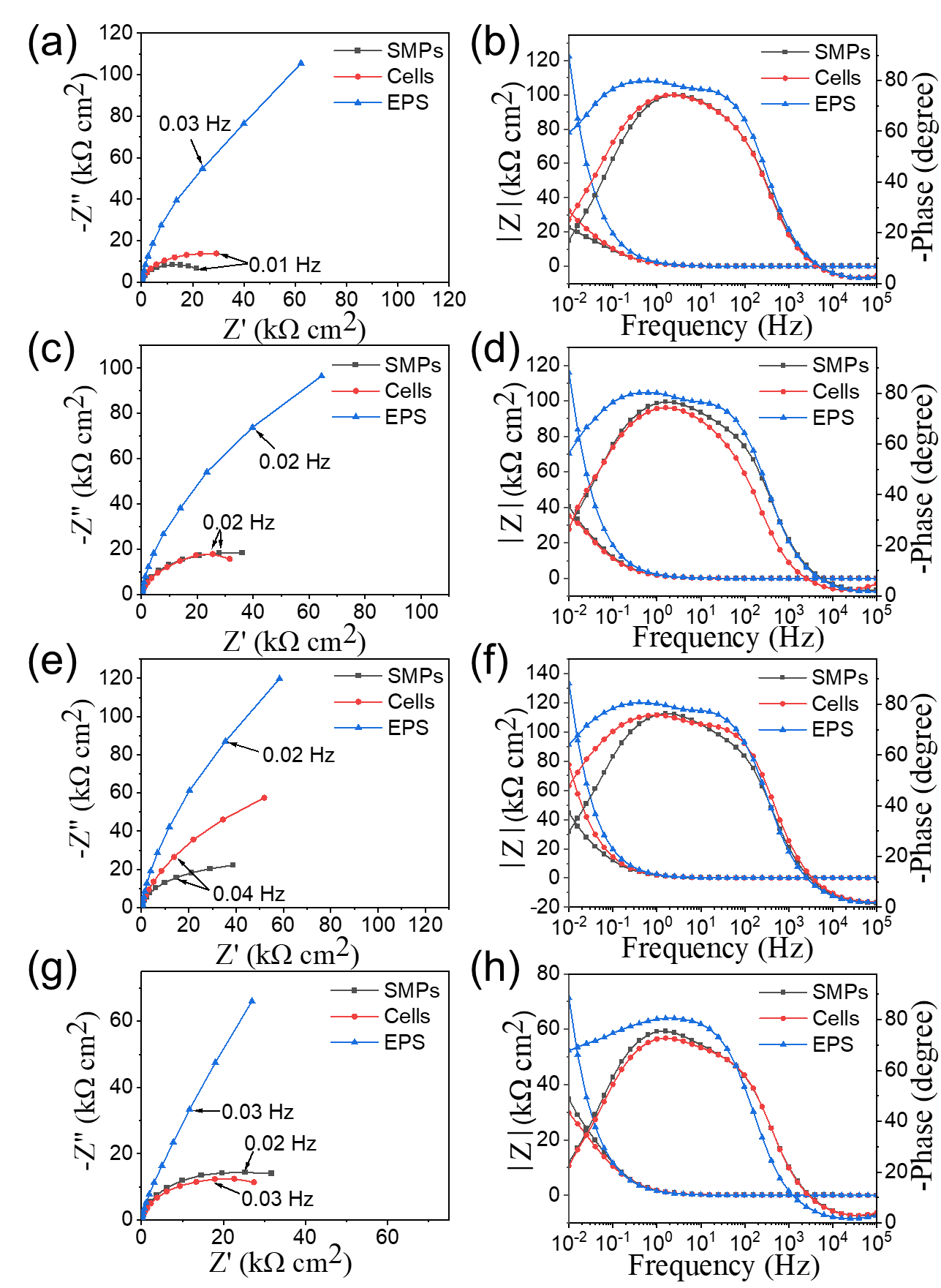
**

**Fig. S5.** The Nyquist and Bode plots of the B30 Cu–Ni alloy after immersion in *B. subtilis* biomass systems for different times: (a, b) 1 d, (c, d) 3 d, (e, f) 7 d, and (g, h) 14 d.

**Table S2** EIS fitting parameters of the B30 Cu–Ni alloy immersed in different *B. subtilis* biomass systems.

|  | *t*  (d) | *R*_s_  (Ω·cm^2^) | *R*_ct_  (Ω·cm^2^) | *Q*_dl_ ×10^−5^  (F·cm^−2^) | *n*_dl_ | *R*_f_  (Ω·cm^2^) | *Q*_f_ ×10^−4^  (F·cm^−2^) | *n*_f_ |
| --- | --- | --- | --- | --- | --- | --- | --- | --- |
| SMPs | 1 | 12.32±1.11 | (2.37±0.45) ×10^4^ | 7.20±2.12 | 0.77±0.01 | 31.32±10.15 | 0.55±0.24 | 0.92±0.08 |
|  | 3 | 11.57±2.45 | (4.66±0.50) ×10^4^ | 4.54±0.72 | 0.79±0.09 | 75.60±18.57 | 0.69±0.43 | 0.89±0.03 |
|  | 7 | 10.97±1.32 | (5.00±0.27) ×10^4^ | 0.84±0.18 | 0.92±0.05 | (5.94±0.65) ×10^2^ | 1.06±0.59 | 0.85±0.05 |
|  | 14 | 12.52±1.58 | (3.71±0.43) ×10^4^ | 0.44±0.26 | 0.95±0.02 | (1.53±0.76) ×10^3^ | 1.11±0.56 | 0.84±0.06 |
| Cells | 1 | 13.48±1.70 | (3.75±0.48) ×10^4^ | 6.90±3.51 | 0.74±0.03 | 43.22±13.38 | 0.58±0.42 | 0.91±0.08 |
|  | 3 | 13.74±0.87 | (4.15±0.52) ×10^4^ | 1.20±0.68 | 0.95±0.04 | (8.82±0.77) ×10^2^ | 1.02±0.45 | 0.84±0.27 |
|  | 7 | 10.31±2.09 | (1.39±0.29) ×10^5^ | 1.08±0.11 | 0.91±0.15 | (2.72±0.54) ×10^3^ | 0.90±0.27 | 0.86±0.11 |
|  | 14 | 13.22±0.84 | (3.20±0.98) ×10^4^ | 0.83±0.07 | 0.90±0.08 | (1.24±0.24) ×10^3^ | 1.13±0.07 | 0.82±0.07 |
| EPS | 1 | 12.41±1.54 | (2.51±0.18) ×10^5^ | 0.29±0.14 | 0.93±0.07 | (1.43±0.39) ×10^4^ | 0.77±0.06 | 0.89±0.13 |
|  | 3 | 11.98±1.15 | (2.54±0.94) ×10^5^ | 0.23±0.15 | 0.94±0.05 | (1.08±0.56) ×10^4^ | 0.80±0.11 | 0.89±0.22 |
|  | 7 | 11.76±1.26 | (4.16±0.40) ×10^5^ | 0.18±0.08 | 0.94±0.05 | (2.29±0.44) ×10^4^ | 0.77±0.07 | 0.89±0.05 |
|  | 14 | 13.23±0.81 | (2.24±0.64) ×10^5^ | 3.46±1.11 | 0.90±0.08 | (1.55±0.13) ×10^4^ | 1.12±0.13 | 0.91±0.08 |

**Table S3** Fitting results of the potentiodynamic polarization curves of B30 Cu–Ni alloy in different *B. subtilis* biomass systems.

|  | *E*_cor_ (V) | *i*_cor_ (A/cm^2^) | *β*_a_ (V/dec) | *β*_c_ (V/dec) |
| --- | --- | --- | --- | --- |
| SMPs | −0.58±0.02 | (1.32±0.10)×10^−7^ | 0.154±0.008 | 0.139±0.010 |
| Cells | −0.60±0.03 | (2.45±0.08)×10^−7^ | 0.172±0.005 | 0.109±0.004 |
| EPS | −0.49±0.02 | (9.05±0.16)×10^−8^ | 0.244±0.020 | 0.176±0.014 |
